# Surface‐Bound Superoxide Radical‐Mediated Photo‐Fenton Mineralization of Ciprofloxacin on Fe‐Sillenite Nanosheets

**DOI:** 10.1002/advs.202522479

**Published:** 2026-03-16

**Authors:** Wenting Qiu, Ji Liu, Hengjun Shang, Yaning Zhang, Shuai Dou, Jing Xu, Ying Zhang, Yang Lou, Yongfa Zhu, Chengsi Pan

**Affiliations:** ^1^ International Joint Research Center for Photoresponsive Molecules and Materials Jiangnan University Wuxi Jiangsu China; ^2^ School of Chemical and Materials Engineering Jiangnan University Wuxi Jiangsu China; ^3^ School of Food Science and Technology Jiangnan University Wuxi Jiangsu China; ^4^ Department of Chemistry Tsinghua University Beijing China

**Keywords:** fenton, H_2_O_2_, mineralization, sillenite, superoxide

## Abstract

Refractory antibiotics frequently generate highly toxic and persistent intermediates during advanced oxidation processes, which severely limit their safe and complete removal. Recent studies show that the fluoroquinolone antibiotic ciprofloxacin (CIP) commonly accumulates quinone‐imine intermediates in Fenton systems, leading to reduced mineralization efficiency and increased toxicity risks. In this work, iron‐based sillenite (Bi_12_FeO_20_) nanosheets are developed to construct an efficient photo‐Fenton system, achieving rapid degradation (94.3% in 8 min) and deep mineralization (TOC 93.8% in 2 h). Comprehensive intermediate‐state identification and theoretical calculations reveal that Bi_12_FeO_20_ stabilizes surface‐bound superoxide radicals (·O_2_^−^) with significantly enhanced reducing capability, whose HOMO energy levels are markedly higher than those of free·O_2_^−^. These surface‐bound radicals efficiently inject electrons into the LUMO of quinone‐imine intermediates, triggering aromatic ring‐opening reduction followed by progressive oxidation, ultimately enabling complete structural destruction and toxicity elimination. Compared with conventional pathways dominated by free ROS, this study establishes a surface‐bound radical‐driven “interfacial directed reduction–oxidation coupled mineralization mechanism.” This work highlights the structural advantages of sillenite for interfacial radical regulation and the removal of refractory intermediates, offering a new materials strategy for designing Fenton catalysts and achieving safe antibiotic treatment in aquatic environments.

## Graphical abstract

This study systematically compares the degradation efficiency of CIP in a H_2_O_2_‐alone system and a novel Bi_12_FeO_20_‐H_2_O_2_ photo‐Fenton system. It reveals for the first time the presence of a quinone‐imine intermediate in the H_2_O_2_‐alone system, which exhibits toxicity comparable to CIP. This toxic intermediate is effectively degraded in the Bi_12_FeO_20_‐H_2_O_2_ system through a reduction pathway mediated by Fe‐atom surface‐adsorbed·O_2_.

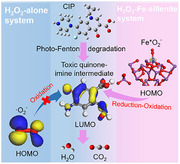

## 1 Introduction

Fluoroquinolone antibiotics, such as ciprofloxacin (CIP), are widely detected in aquatic environments due to their high structural stability, poor biodegradability, and potential risks associated with the spread of antimicrobial resistance, making them representative refractory organic pollutants [1, 2]. Although existing treatment technologies, including adsorption [3], biological processes [4], and UV/chlorine oxidation [5], can achieve rapid removal of parent compounds, they often result in the formation of structurally complex and more toxic transformation products (quinoline derivatives [6], defluorinated products [7], and piperazine ring‐cleavage products) [8], hindering complete detoxification. In Fenton and photo‐Fenton systems, despite the strong oxidative capability of hydroxyl radicals (·OH), the degradation of CIP frequently stalls at nitrogen‐containing aromatic intermediates [9, 10], Based on this understanding, the present study further identifies and elucidates the formation mechanism of refractory intermediates during the degradation of CIP.

To address the accumulation of these persistent intermediates, Fenton and photo‐Fenton processes activate H_2_O_2_ to generate reactive oxygen species (ROS), including ·OH and superoxide radicals (·O_2_^−^), enabling the initial degradation of antibiotics [11, 12]. However, their deep mineralization performance remains intrinsically constrained. On the one hand, free ·O_2_^−^ exhibits insufficient reducing power to effectively disrupt the highly delocalized electronic structures of stubborn intermediates [13]. On the other hand, free ROS possess short lifetimes and high diffusivity, making them readily quenched by water matrix components such as Cl^−^, CO_3_^2−^, and natural organic matter, thereby significantly reducing the effective concentration of reactive species [14]. These limitations collectively lead to the persistent accumulation of refractory intermediates and even toxicity rebound after treatment [12]. Consequently, achieving complete detoxification requires a fundamental shift from free‐radical‐dominated pathways toward more stable and selective interfacial reactive species.

Recent studies suggest that surface‐bound radicals confined on catalyst surfaces exhibit prolonged lifetimes, higher local concentrations, and superior interfacial electron‐transfer capabilities, enabling them to overcome the intrinsic limitations of free radicals [15, 16, 17, 18, 19]. In particular, surface‐bound ·O_2_^−^, whose electronic structure is regulated by confinement effects and metal‐oxygen coordination environments, often displays enhanced reducing properties and may induce directional attacks on electron‐rich aromatic structures [20, 21]. Nevertheless, the role of surface‐bound ·O_2_^−^ in regulating refractory intermediates during antibiotic degradation and its contribution to deep mineralization remain insufficiently understood.

Sillenite‐structured materials (Bi_12_MO_20_) have recently attracted increasing attention due to their non‐centrosymmetric crystal structures and pronounced dipole moments, which endow them with unique advantages in interfacial charge regulation and stabilization of reactive species [22, 23, 24, 25]. Among them, iron‐based sillenite Bi_12_FeO_20_ features Fe‐O tetrahedral units, enabling flexible modulation of the electronic structure while maintaining high structural stability, with negligible Fe leaching. These characteristics provide a favorable structural basis for stabilizing surface‐bound ·O_2_^−^ species and regulating interfacial electron transfer. However, the role of Bi_12_FeO_20_ in Fenton systems, particularly in controlling refractory antibiotic intermediates, has not yet been systematically investigated. Against this background, the present study employs Bi_12_FeO_20_ nanosheets as a model catalyst to construct a photo‐Fenton system and elucidates the critical role of surface‐bound ·O_2_^−^ in regulating persistent intermediates and achieving deep mineralization, thereby offering new insights into the design of Fenton catalysts and interfacial radical chemistry.

## 2 Results and Discussion

### 2.1 Morphology and Structure of the Prepared Bi_12_FeO_20_ Sillenite Nanosheets

**FIGURE 1:**
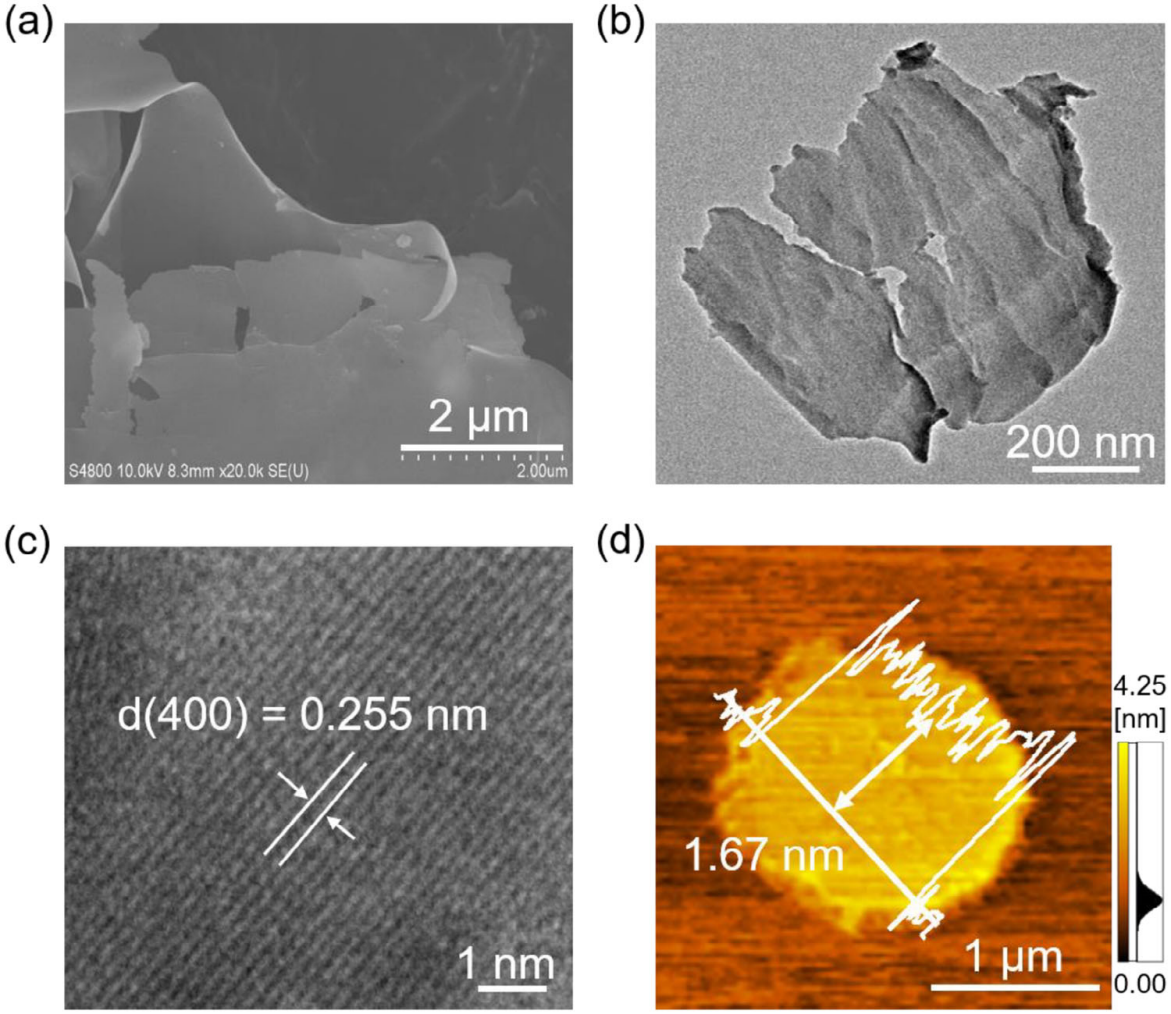
(a) SEM, (b) TEM, (c) HRTEM, and (d) AFM of Bi_12_FeO_20_ nanosheets.

Figure 1a,b show the scanning electron microscope (SEM) and transmission electron microscopy (TEM) images of the prepared Bi_12_FeO_20_. Bi_12_FeO_20_ exhibits a nanosheet structure (size: 0.5–4.5 µm). High‐resolution transmission electron microscopy (HRTEM) in Figure 1c reveals that the lattice spacing of Bi_12_FeO_20_ is 0.255 nm, which matches the interplanar distance of the [400] planes in sillenites. This suggests that the Bi_12_FeO_20_ sillenite nanosheets grow along the [400] direction. A similar growth direction is observed for other sillenite nanosheets, such as Bi_2_O_3_ and Bi_12_CoO_20_.[26, 27]. Figure 1d shows the atomic force microscope (AFM) image of the prepared Bi_12_FeO_20_ nanosheets. The thickness of the Bi_12_FeO_20_ nanosheets is about 1.67 nm, composed of 6 layers of stacked [400] planes.

**FIGURE 2:**
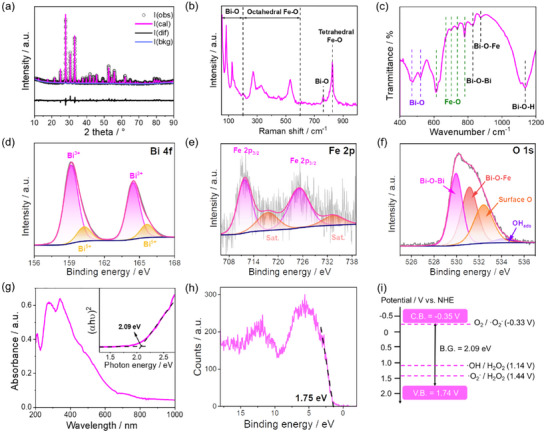
(a) XRD refinement, (b) Raman spectrum, (c) FTIR spectrum, (d) XPS narrow scan of the Bi 4f spectra and the fitting curves, (e) XPS narrow scan of the Fe 2p spectra and the fitting curves, (f) XPS narrow scan of the O 1s spectra and the fitting curves, (g) UV‐DRS spectrum, (h) XPS valence band spectrum and (d) Potential diagram of the prepared Bi_12_FeO_20_ nanosheet. Figure 2g inset is the concurrent Tauc plot.

X‐Ray diffraction (XRD) analysis confirms the crystal structure of the synthesized Bi_12_FeO_20_ nanosheets. The XRD pattern aligns with the standard Bi─Fe─O reference, showing no peak shifts or extra peaks, indicating a pure sillenite structure free of impurities (Figure S1). To further clarify the structure, in Figure 2a, structural refinement is performed using the Rietveld method, with detailed parameters listed in Table S1. The displays experimental data points (circles), superimposed with the refined profile (pink), background signal (blue), and residual curve (black). The high consistency between experimental and calculated patterns is evidenced by minimal residual fluctuations. Reliability factors quantifying refinement quality yield R_wp_ = 9.65% and χ^2^ = 2.47, similar to the reported data in established sillenite systems, including Bi_12_ZnO_20_ (R_wp_ = 24.15%, χ^2^ = 3.22) and Bi_12_TiO_20_ (R_wp_ = 33.3%, χ^2^ = 5.02) [23, 25]. XRD analysis reveals a cubic cell parameter of 10.195 Å, exhibiting a 0.77% expansion compared to the theoretical 10.118 Å. Site occupancy refinement identifies Fe^3+^ distribution across tetrahedral (0.587) and octahedral (0.051) positions. The distribution closely matches the results obtained from ICP‐OES analysis, which reveals a total iron content of 0.978. This value is comparable to the refined Fe content (1.199), verifying the refined results. The occupation of Fe at tetrahedral sites is larger than the theoretical one (0.5), indicating Fe^3+^ fully occupies all tetrahedral positions.

SEM‐EDX analysis of the Bi_12_FeO_20_ nanosheet shows a uniform distribution of Bi, Fe, and O, with atomic contents of 36.8% Bi and 2.9% Fe, closely matching the theoretical values (36.4% Bi, 3.0% Fe) (Figure S2). Combined with XRD results, this confirms the successful synthesis of pure‐phase Bi_12_FeO_20_ nanosheets.

Raman, FTIR, and XPS analyses are performed further to investigate the material's structure (Figure 2b–f). The Raman spectrum reveals distinct vibrational features across three characteristic regions: 50–200, 200–600, and 600–800 cm^−1^. These spectral bands are attributed to the Bi─O and Fe─O stretching vibrations in the octahedral coordination environment, and the Bi─O and Fe─O bonds associated with the tetrahedral sites in the sillenite lattice, respectively [27, 28, 29]. This indicates that for the sample, Fe and Bi occupy both tetrahedral and octahedral sites. The FTIR spectrum exhibits five distinct absorption bands centered at 400–560, 560–800, 827, 875, and 1138 cm^−1^. These spectral features are attributed to vibrational modes corresponding to Bi─O bonds, Fe─O stretching, Bi─O─Bi linkages, Bi─O─Fe interactions, and Bi─OH groups on the surface, respectively [28, 30]. Raman and FTIR spectra both confirm the presence of Bi─O and Fe─O stretching vibrations in tetrahedral coordination, as well as Fe─O stretching in octahedral coordination. This behavior is consistent with our previous findings regarding the introduction of Co into the sillenite lattice [27].

Additionally, XPS shows Bi 4f binding energies at 159.2 and 164.5 eV (Bi^3+^) and 160.3 and 165.6 eV (Bi^5+^), aligning with reported values [22, 30]. The Fe 2p spectrum displays main peaks for Fe 2p_3/2_ and Fe 2p_1/2_ at 711.5 and 724.8 eV, respectively, along with satellite peaks at 716.9 eV (Fe 2p_3/2_) and 732.7 eV (Fe 2p_1/2_), which are characteristic peaks of Fe^3+^ [31, 32]. The present O 1s XPS spectrum, deconvoluted into four components with binding energies of 529.9, 531.2, 532.4, and 534.2 eV. Based on previous studies, these components are identified as Bi─O─Bi, Bi─O─Fe, surface oxygen, and hydroxyl groups, respectively [32, 33]. The XPS analysis of Bi, Fe, and O elements confirms the effective incorporation of Bi^5+^ and Fe^3+^ into the tetrahedral positions of the sillenite structure. Based on the combined Raman, FTIR, XPS, and EDX results, the molecular formula of Bi_12_FeO_20_ is determined to be (Bi^III^_23_Fe^III^)(Bi^V^Fe^III^O_8_)O_32_, consistent with charge balance considerations. These findings align with previous observations in the literature [27]. In addition, thermogravimetric analysis is also conducted on the Bi_12_FeO_20_ catalyst, and the results demonstrate its good thermal stability (Figure S3).

The band structure of the synthesized Bi_12_FeO_20_ sillenite nanosheets is further investigated. As shown in the UV‐DRS spectrum (Figure 2g), the Tauc plot reveals a bandgap of 2.09 eV. Mott–Schottky curve (Figure S4) and XPS valence band (Figure 2h) analyses position the Fermi level at −0.22 eV vs. NHE and the valence band edge at 1.74 eV vs. NHE, yielding a conduction band edge at −0.35 eV vs. NHE (Figure 2i). This band structure enables Bi_12_FeO_20_ nanosheets to activate H_2_O_2_, generating the common ROS for efficient CIP degradation.

### 2.2 Photo‐Fenton Removal of CIP and Its Recalcitrant Degradation Intermediates in the H_2_O_2_‐Alone System

**FIGURE 3:**
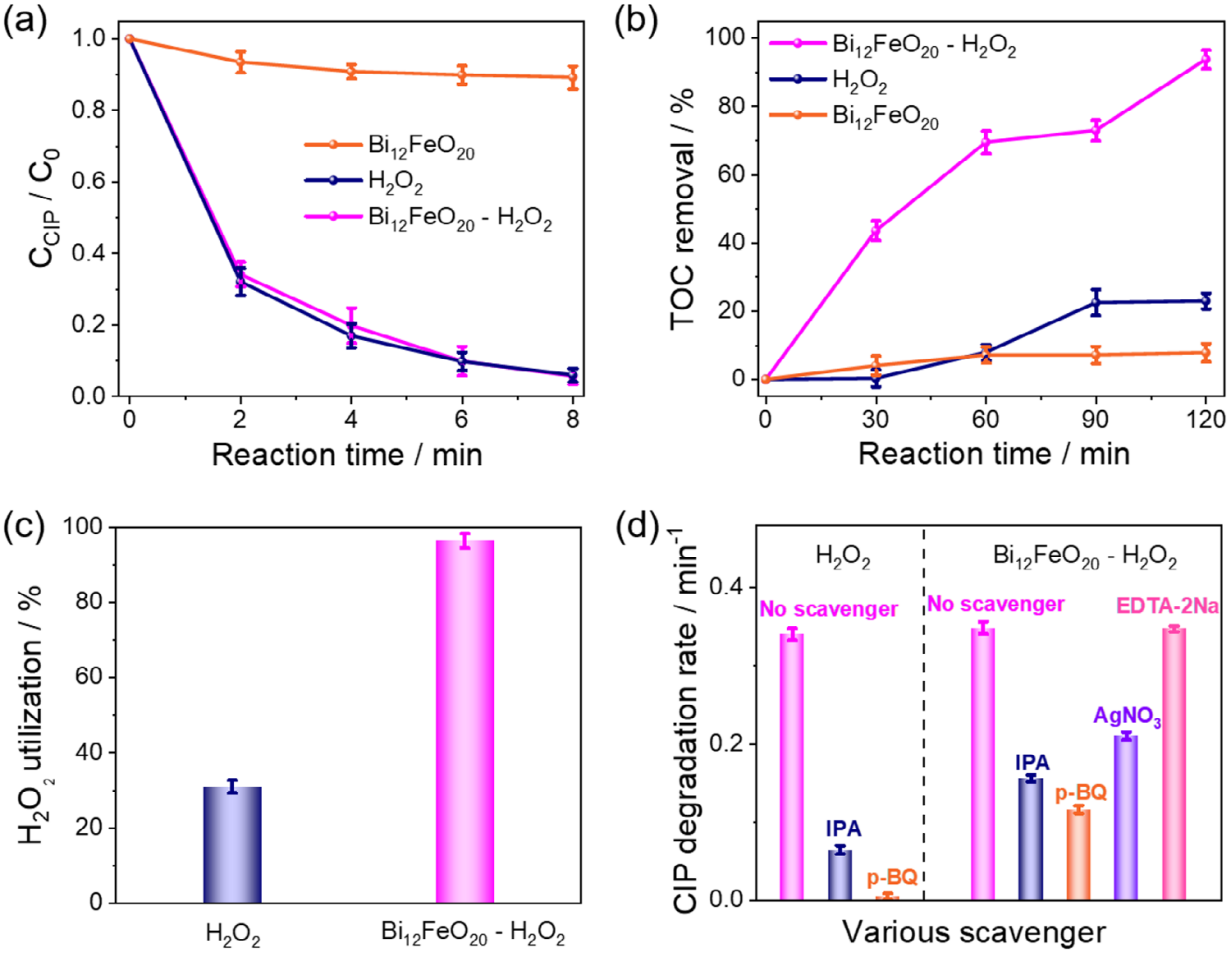
(a) The Photo‐Fenton degradation performance of CIP in the Bi_12_FeO_20_‐alone, H_2_O_2_‐alone, and Bi_12_FeO_20_‐H_2_O_2_ systems. (b) The TOC removal of CIP in the Bi_12_FeO_20_‐alone, H_2_O_2_‐alone, and Bi_12_FeO_20_‐H_2_O_2_ systems. (c) The H_2_O_2_ utilization efficiency during the CIP degradation in the H_2_O_2_‐alone and Bi_12_FeO_20_‐H_2_O_2_ systems. (d) The CIP degradation rate with different ROS scavengers in the H_2_O_2_‐alone and Bi_12_FeO_20_‐H_2_O_2_ systems. Degradation conditions: [CIP] = 10 mg·L^−1^, [Bi_12_FeO_20_] = 0.5 g·L^−1^, [H_2_O_2_] = 2 mm, [IPA] = 5 mm, [p‐BQ] = [AgNO_3_] = [EDTA‐2Na] = 1 mm.

We then investigate the photo‐Fenton degradation activity of Bi_12_FeO_20_. Figure 3a presents the time‐dependent degradation curves of CIP in the Bi_12_FeO_20_‐H_2_O_2_ system. The adsorption of CIP on Bi_12_FeO_20_, the degradation of CIP by H_2_O_2_ in the dark, and the direct photolysis of CIP under light are examined. All these processes exhibit negligible effects on the subsequent photocatalytic degradation (Figure S5). Figure S6 shows the specific surface area of the Bi_12_FeO_20_ nanosheets. The measured value is 27.01 m^2^ g^−1^, indicating that adsorption is not the primary factor responsible for the observed differences in catalytic activity. Under optimized conditions (Figure S7), the Bi_12_FeO_20_‐H_2_O_2_ system achieves rapid CIP degradation, with a degradation efficiency of 94.3% within 8 min, significantly higher than the 10.8% in the Bi_12_FeO_20_‐alone system. However, when compared to the H_2_O_2_‐alone system, which exhibits a degradation rate of 94.0%, no significant improvement is observed. This raises the question of whether the degradation is primarily driven by H_2_O_2_ itself, with minimal contribution from the Bi_12_FeO_20_ catalyst. In addition, CIP at low concentrations (1 ppm and 5 ppm) is also tested, and the results show that the Bi_12_FeO_20_‐H_2_O_2_ system achieves degradation efficiencies of 99.4% and 97.1%, respectively, within 8 min (Figure S8).

To further clarify this, the total organic carbon (TOC) removal is evaluated in the Bi_12_FeO_20_‐alone, H_2_O_2_‐alone, and Bi_12_FeO_20_‐H_2_O_2_ systems (Figure 3b). In the Bi_12_FeO_20_‐alone system, the TOC removal percentage of CIP is 7.9% after 2 h of degradation, while in the H_2_O_2_‐alone system, it reaches 23.0%, indicating only partial mineralization of CIP. In contrast, the Bi_12_FeO_20_‐H_2_O_2_ system achieves a significantly higher TOC removal percentage of 93.8% within 2 h. While both the H_2_O_2_‐alone and Bi_12_FeO_20_‐H_2_O_2_ systems show similar degradation rates, their TOC removal efficiencies differ significantly. This indicates that Bi_12_FeO_20_ enhances mineralization, underscoring its role in driving complete degradation. Therefore, we apply the Bi_12_FeO_20_ catalyst in a microbubble system for CIP degradation, achieving a CIP removal efficiency of 90.1% within 60 min. Meanwhile, the Bi_12_FeO_20_ catalyst is used to activate PMS, resulting in a CIP degradation efficiency of 93.1% within 8 min. These results demonstrate that the Bi_12_FeO_20_ catalyst exhibits excellent performance for CIP degradation across different reaction systems (Figure S9).

We analyze H_2_O_2_ utilization efficiency (η), defined as the ratio of stoichiometric to actual H_2_O_2_ consumption (Text S4). As shown in Figure 3c, Figure S10, and Table S2, the η value of the Bi_12_FeO_20_‐H_2_O_2_ system (96.4%) is approximately 3.2 times higher than that of the H_2_O_2_‐alone system (30.1%). This enhanced efficiency explains the significantly higher TOC removal in the Bi_12_FeO_20_‐H_2_O_2_ system.

To identify the ROS in CIP degradation, radical quenching experiments are performed using isopropyl alcohol (IPA), p‐benzoquinone (p‐BQ), silver nitrate (AgNO_3_), and ethylenediaminetetraacetic acid disodium salt (EDTA‐2Na) as scavengers for ·OH, ·O_2_^−^, electron (e^−^), and hole (h^+^), respectively [34, 35]. In the H_2_O_2_‐alone system (Figure 3d; Figure S11a), the rate constant (0.341 min^−1^) significantly decreases to 0.064 min^−1^ (·OH scavenged) and 0.005 min^−^^1^ (·O_2_^−^ scavenged). Similarly, in the Bi_12_FeO_20_‐H_2_O_2_ system (Figure 3d; Figure S11b), the initial degradation rate constant (0.349 min^−^^1^) decreases to 0.156 min^−1^ (·OH scavenged), 0.116 min^−1^ (·O_2_^−^ scavenged), and 0.210 min^−1^ (e^−^ scavenged), while h^+^ scavenging shows less impact (0.348 min^−1^). The application of L‐Histidine (L‐His) for singlet oxygen trapping does not demonstrate any inhibitory impact on CIP degradation (Figure S11c). Based on the results of radical quenching experiments and electron paramagnetic resonance (EPR) analysis (Figure S12), we propose that ·O_2_^−^ plays a pivotal role in the Bi_12_FeO_20_‐H_2_O_2_ system. Then we further investigate its generation mechanism. Ar purging (Figure S13) shows ·O_2_^−^ is generated from H_2_O_2_ decomposition, not dissolved oxygen. The rotating ring‐disk electrode (RRDE) tests (Figure S14) confirm that ·O_2_^−^ is produced via single‐electron reduction of H_2_O_2_ in the Bi_12_FeO_20_‐H_2_O_2_ system.

**FIGURE 4:**
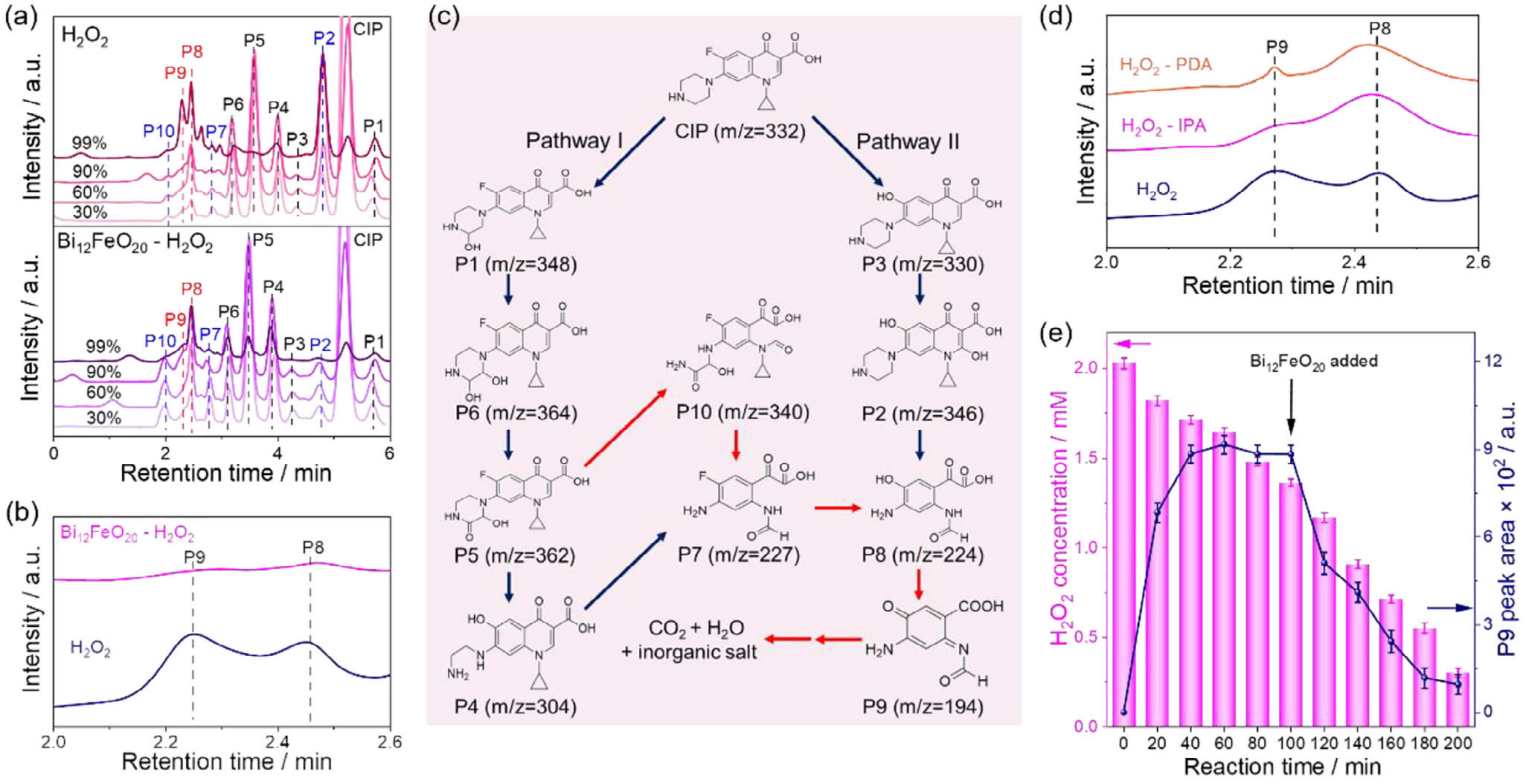
(a) The HPLC spectra of CIP removal by 30% (2 min), 60% (4 min), 90% (8 min), and 99% (20 min) in the H_2_O_2_‐alone system, and the CIP removal by 30% (2 min), 60% (4 min), 90% (6 min), and 99% (20 min) in the Bi_12_FeO_20_‐H_2_O_2_ system. (b) The HPLC spectra with extended degradation time to 90 min for H_2_O_2_‐alone and Bi_12_FeO_20_‐H_2_O_2_ systems. (c) The possible degradation pathway of CIP in the H_2_O_2_‐alone and Bi_12_FeO_20_‐H_2_O_2_ system. (d) The HPLC spectra of CIP degradation at the time when the CIP peak is completely removed in the H_2_O_2_‐alone system with different capture reagents. (e) The changes of the P9 intermediate and H_2_O_2_ concentration depending on the time variation in the H_2_O_2_ system before and after the addition of Bi_12_FeO_20_. Degradation conditions: [CIP] = 10 mg·L^−1^, [Bi_12_FeO_20_] = 0.5 g·L^−1^, [H_2_O_2_] = 2 mm, [IPA] = 5 mm, [PDA] = 10 ppm.

To illustrate the intermediates produced during the CIP degradation process in the H_2_O_2_‐alone and Bi_12_FeO_20_‐H_2_O_2_ systems, we compare a series of high‐performance liquid chromatography spectra (HPLC) with the same percentage of CIP removal in Figure 4a. However, in other iron‐based Fenton systems under the same reaction conditions (such as the FeS_2_‐H_2_O_2_ system and Fe_3_O_4_‐H_2_O_2_ system), a weaker removal efficiency of CIP is also observed (Figure S15). We focus on the H_2_O_2_‐alone system due to its minimal Fe‐leaching and rapid H_2_O_2_ decomposition, which allows clear identification of intermediate and facilitates comparison with the Bi_12_FeO_20_‐H_2_O_2_ system.

The analysis shows that while the two systems produce similar intermediates, the amounts that accumulate vary significantly. Based on their retention time, these intermediates are labeled as P1‐P10 in the figures. Notably, distinct differences are observed in the peak areas of P2 (4.75 min), P7 (2.81 min), and P10 (2.00 min) intermediates produced by the two systems (Figure S16). Specifically, at 90% CIP removal, the H_2_O_2_‐alone system shows 7.1 times more P2, while the Bi_12_FeO_20_‐H_2_O_2_ system generates 3.5 times more P7 and 19.7 times more P10, suggesting different degradation pathways.

Additionally, when we extend the reaction time to 90 min in Figure 4b, we surprisingly observe that only P8 (2.45 min) and P9 (2.33 min) intermediates remain in the H_2_O_2_‐alone system, while the peaks of other intermediates (P1‐P7 and P10) become almost negligible. In contrast, the intermediates in the Bi_12_FeO_20_‐H_2_O_2_ system nearly disappear. The peak area differences of P8 and P9 between the two systems reach 4.4 times and 11.6 times, respectively. This indicates that, in the H_2_O_2_‐alone system, the P8 intermediate likely converts into the P9 intermediate over time, while the P9 intermediate cannot be further transformed, leading to its accumulation. Therefore, we speculate that the P9 intermediate is the key factor hindering the complete mineralization of CIP in the H_2_O_2_‐alone system. Notably, in the H_2_O_2_‐alone system, extending the reaction time to 8 h does not lead to further reduction of the P9 intermediate, nor does increasing the H_2_O_2_ concentration to 5 mM; instead, it results in an accumulation of the P8 intermediate (Figure S17), indicating that the degradation disparity between the H_2_O_2_‐alone and Bi_12_FeO_20_‐H_2_O_2_ systems stems from thermodynamic rather than kinetic limitations.

The degradation pathway of CIP and the formation of P9 intermediate are comprehensively investigated using ultra‐performance liquid chromatography‐quadrupole time‐of‐flight mass spectrometry (UPLC‐Q‐TOF‐MS). The degradation process proceeds through two distinct pathways, as illustrated in Figure 4c, Figure S18, and Table S3. In Pathway I, the initial step involves hydroxylation of the piperazine ring, leading to the formation of P1 (m/z = 348). Subsequent hydroxylation of P1 converts P6 (m/z = 364), which undergoes oxidation to form P5 (m/z = 362). The degradation continues with the cleavage of both the piperazine and pyridone rings, yielding P4 (m/z = 306) and P10 (m/z = 340). Further degradation involves the removal of the piperazine ring and cyclopropyl group, resulting in the formation of P7 (m/z = 227). Finally, the substitution of fluorine with a hydroxyl group produces P8 (m/z = 224). Pathway II initiates with the defluorination of the quinolone ring, forming P3 (m/z = 330), which is subsequently hydroxylated to yield P2 (m/z = 346). The degradation proceeds with the cleavage of the piperazine ring, pyridinone ring, and cyclopropyl group, ultimately producing P8, which contains both imide and phenol functional groups. Notably, P8 is an intermediate produced in both degradation pathways. This intermediate is further oxidized to form the more stable and recalcitrant intermediate P9 (m/z = 194), characterized by a quinone‐imine structure. The elucidation of these pathways provides critical insights into the degradation mechanisms of CIP and highlights the formation of P9 recalcitrant intermediates, which may pose environmental challenges due to their resistance to further degradation.

Based on the combined analysis of HPLC and LC‐MS, it is revealed that both the H_2_O_2_‐alone and Bi_12_FeO_20_‐H_2_O_2_ systems simultaneously follow Pathway I and Pathway II during CIP degradation. The key distinction lies in the fact that the H_2_O_2_‐alone system is unable to proceed with further degradation after reaching the quinone‐imine intermediate (P9), while the Bi_12_FeO_20_‐H_2_O_2_ system successfully facilitates subsequent degradation steps. This fundamental difference in the degradation capability ultimately accounts for the observed divergence in TOC removal efficiency between the two systems. In addition, our experimental results reveal that other fluoroquinolone contaminants, including ofloxacin (OFX) and norfloxacin (NFX), consistently produce the P9 intermediate during 8 h reactions in the H_2_O_2_‐alone system, yet remain resistant to subsequent degradation processes (Figure S19).

The formation of P9 during the CIP degradation process is further investigated. In the H_2_O_2_‐alone system, polydopamine (PDA) nanoparticles and IPA are independently introduced to capture the ROS (·O_2_^−^ and ·OH) involved in the degradation of CIP [36]. The changes in the HPLC peak area of P9 are analyzed. As shown in Figure 4d, after 90 min reaction, the capture of either ·O_2_^−^ or ·OH results in a 0.5 times and 1.7 times increase in the peak area of the P8 intermediate, respectively, while the peak area of the P9 intermediate decreases by 85% and 56%. Notably, the capture of either ·O_2_^−^ or ·OH alone prevents the formation of P9. However, when both ·O_2_^−^ and ·OH are present simultaneously, the peak area of P9 reaches its highest compared to scenarios where only one of the ROS is captured. This observation suggests that the combined action of ·O_2_^−^ and ·OH synergistically promotes the conversion of the P8 intermediate into the P9 intermediate.

To investigate the role of Bi_12_FeO_20_ in the degradation of the P9 intermediate, Bi_12_FeO_20_ is introduced into a solution where CIP has been completely removed in the H_2_O_2_‐alone system. As shown in Figure 4e, the CIP solution is initially degraded in a 2.03 mm H_2_O_2_ system, and the peak area of the P9 intermediate increases gradually with reaction time. After 100 min, the P9 intermediate peak area stabilizes, and the H_2_O_2_ concentration at this point is measured to be 1.36 mm. Subsequently, the Bi_12_FeO_20_ catalyst is added to the P9‐containing solution. Within just 20 min, the P9 intermediate peak area decreases by 42.1%. After continuing the reaction for 200 min, the P9 intermediate is degraded by 89.2%. These results demonstrate that while the P9 intermediate persists in the H_2_O_2_‐alone system, its degradation is significantly enhanced by the Bi_12_FeO_20_ catalyst.

### 2.3 Toxicity Evaluation of Quinone‐Imine Intermediates (P9) and Its Removal by Surface‐Bounded ·O_2_^−^ In the Bi_12_FeO_20_‐H_2_O_2_ System

**FIGURE 5:**
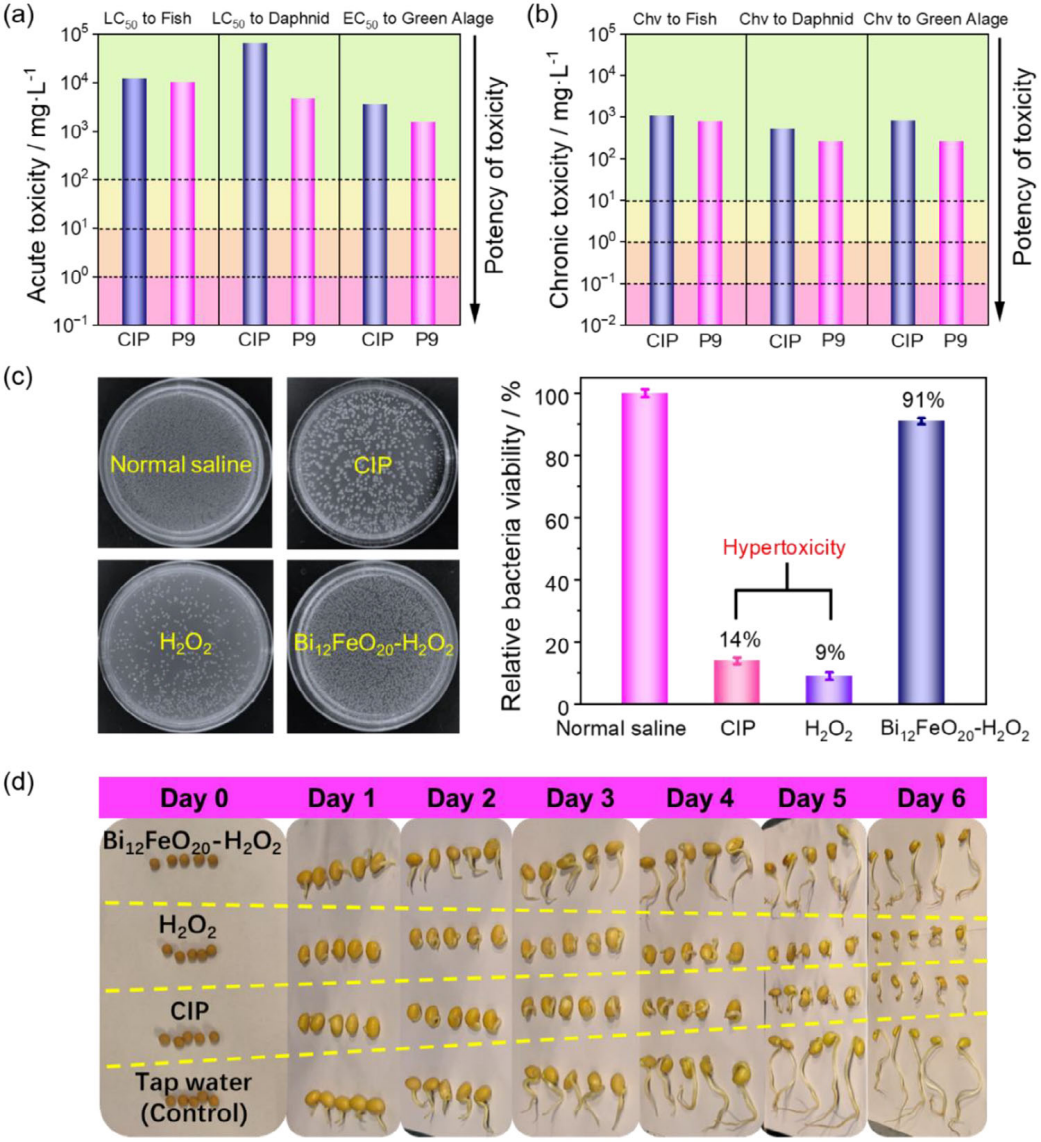
(a) The acute and (b) chronic toxicity of CIP and quinone‐imine (P9) intermediates to fish, daphnid, and green algae. The ECOSAR software performs the toxicity prediction. (c) The colony images and the relative bacteria viability of the toxicity test with Escherichia coli O157 using the untreated and treated 10 ppm CIP solution in the Bi_12_FeO_20_‐H_2_O_2_ and H_2_O_2_‐alone system. The normal saline is used as the control. (d) The growth of soybean sprouts in effluents treated by different methods. The average of soybeans on Day 0 is 0.80*0.86 cm. Reaction conditions: [CIP] = 10 mg·L^−1^, [Bi_12_FeO_20_] = 0.5 g·L^−1^, [H_2_O_2_] = 2 mm. The treatment time for the CIP solution is 2 h, and the residual H_2_O_2_ is treated with MnO_2_.

Based on the above discussion, we conclude that the H_2_O_2_‐alone system generates a stable quinone‐imine intermediate as the final product during CIP degradation, while the Bi_12_FeO_20_‐H_2_O_2_ system directly degrades the quinone‐imine intermediate in the process. Consequently, we perform toxicity assessments on the solutions treated by these two systems to evaluate their environmental implications. The acute and chronic toxicity of CIP and its quinone‐imine intermediate (P9) are evaluated using ECOSAR software (Figure 5a,b; Table S4). The toxicity levels are color‐coded: green indicates not harmful, while pink, orange, and yellow represent highly toxic, toxic, and harmful, respectively. The results reveal that CIP is harmful to fish (LC_50_ = 11.10 mg·L^−1^), not harmful to daphnia (EC_50_ = 12.30 mg·L^−1^), and toxic to green algae (EC_50_ = 4.43 mg·L^−1^). Similarly, the P9 intermediate demonstrates comparable toxicity, being harmful to fish (LC_50_ = 12.90 mg·L^−1^), harmful to daphnia (EC_50_ = 17.71 mg·L^−1^), and toxic to green algae (EC_50_ = 5.93 mg·L^−1^). In addition, the ECOSAR software is used to evaluate the acute and chronic toxicity of the other intermediates (P1‐P8, P10). To verify the accuracy of the results, we also used the T.E.S.T. software to predict the toxicity of CIP and the P9 intermediate to Daphnia magna (48h), and the findings indicate a similarity to the toxicity predicted by the ECOSAR software (Figure S20). Notably, a structurally similar quinone‐imine intermediate has also been identified during the degradation of paracetamol [37]. During CIP degradation in the H_2_O_2_‐alone system, the accumulation of the P9 intermediate increases the overall toxicity in aquatic environments, thereby limiting the potential for effective water purification. In contrast, the Bi_12_FeO_20_‐H_2_O_2_ system facilitates the further mineralization of the quinone‐imine intermediate, significantly reducing the toxicity of the treated CIP wastewater.

We further assess the toxicity of the degradation solution containing the P9 intermediate (H_2_O_2_‐alone system treated) and without the P9 intermediate (Bi_12_FeO_20_‐H_2_O_2_ systems treated) by conducting bacterial incubation experiments, with detailed methodology provided in Text S5. Escherichia coli O157 (*E. coli O157*) serves as the indicator organism for these experiments (Figure 5c). The control sample, cultured in normal saline without CIP, yields a colony count of 7.9 × 10^7^ CFU·mL^−^^1^. Notably, when cultured with untreated CIP, the relative bacterial viability of *E. coli O157* drops significantly to 14% of the control, with a colony count of 1.1 × 10^7^ CFU·mL^−^^1^, demonstrating the strong antibacterial activity of untreated CIP. The colony count in the CIP solution treated with the H_2_O_2_‐alone system remains similarly low at 0.7 × 10^7^ CFU·mL^−^^1^, comparable to that of the untreated CIP solution. This confirms that the toxicity of the persistent quinone‐imine intermediate in the H_2_O_2_‐alone‐treated wastewater is nearly equivalent to that of the untreated CIP, as predicted by the ECOSAR software. In contrast, the relative bacterial viability of *E. coli O157* cultured in the Bi_12_FeO_20_‐H_2_O_2_‐treated water reaches 91% of the control (colony count: 7.2 × 10^7^ CFU·mL^−^^1^), indicating a substantial reduction in toxicity. This result is consistent with the theoretical toxicity profiles of both P9 and CIP. To further verify the reliability of the experimental results, we select 1 ppm and 0.1 ppm concentrations of the CIP solution and its treated solution for parallel testing. The results demonstrate that consistent experimental phenomena are observed under different concentration conditions (Figure S21). Together, these findings demonstrate that the Bi_12_FeO_20_‐H_2_O_2_ system not only effectively degrades CIP but also mineralizes the highly toxic quinone‐imine intermediate, thereby significantly reducing the biological toxicity of the treated water. While the H_2_O_2_‐alone system fails to achieve this level of detoxification.

The toxicity of the degradation solution containing the P9 intermediate is further assessed by employing the soybean as a model crop. As illustrated in Figure 5d, the soybean sprout growth experiment is carried out over six days and included four treatment groups: i) tap water (control), ii) a ciprofloxacin (CIP) solution (10 mg L^−^^1^), iii) degradation effluents containing quinone‐imine (P9) intermediates obtained from the H_2_O_2_‐alone system, and iv) degradation effluents from the Bi_12_FeO_20_‐H_2_O_2_ system, in which quinone‐imine intermediates is completely removed. Detailed growth data are provided in Table S5. The results reveal that in the tap water group (control, normal growth), the average stem length of the soybean sprouts is 14.30 ± 0.6 cm, and the average root length of the soybean sprouts is 6.90 ± 0.1 cm [38]. However, exposure to the 10 ppm CIP solution significantly inhibits the growth of the sprouts, reducing the average stem length to 2.15 ± 0.02 cm, which is a decrease of 84.97% compared to the control group. This outcome confirms the toxic effects of CIP on plant growth. Notably, the degradation solution containing quinone‐imine intermediates exerts an even more pronounced impact on the growth of the sprouts, with the average stem length reduced to 1.75 ± 0.02 cm, which is a decrease of 87.76% compared to the control group. This finding demonstrates that the quinone‐imine intermediates generated during the degradation process by the H_2_O_2_‐alone system possess stronger phytotoxicity than the parent CIP, providing critical evidence for the ecological risks associated with degradation intermediates. In contrast, in the degradation solution treated by the Bi_12_FeO_20_‐H_2_O_2_ system, the average stem length of the soybean sprouts reaches 12.45 ± 0.2 cm, and the average root length of the soybean sprouts is 6.05 ± 0.1 cm. This result indicates that the Bi_12_FeO_20_‐H_2_O_2_ system not only effectively degrades CIP but also significantly reduces the toxicity of the degradation products, consistent with the theoretical results and bacterial incubation experiments.

**FIGURE 6:**
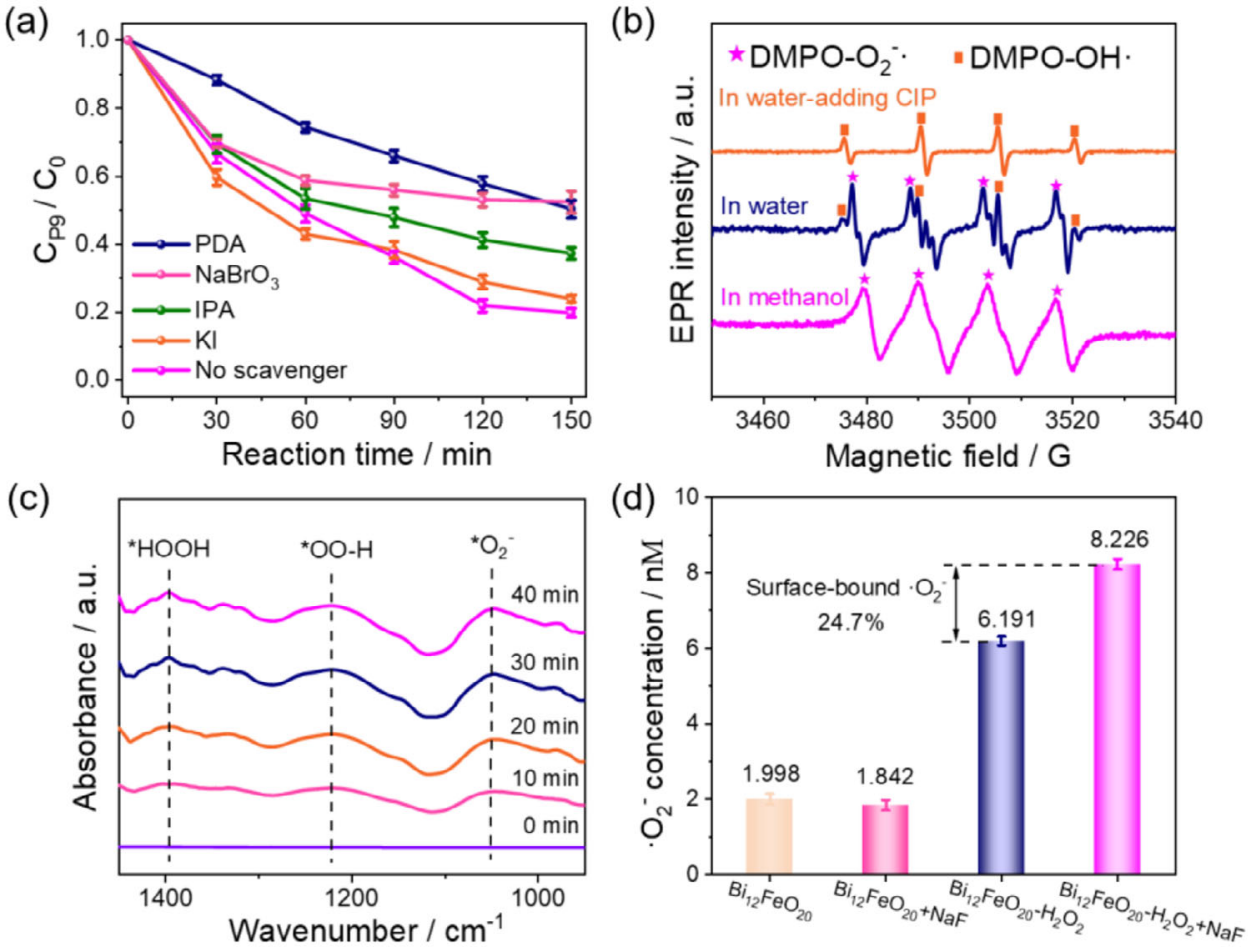
(a) The degradation of the quinone‐imine intermediates (P9) in the Bi_12_FeO_20_‐H_2_O_2_ system with different capture reagents. (b) EPR spectra of the Bi_12_FeO_20_‐H_2_O_2_ system measured in water, in methanol, and in water containing 10 ppm CIP. DMPO is added as a radical capture reagent. (c) In situ FTIR spectra of the Bi_12_FeO_20_‐H_2_O_2_ system recorded as a function of irradiation time. (d) Comparison of the ·O_2_^−^ concentration in the Bi_12_FeO_20_‐alone and Bi_12_FeO_20_‐H_2_O_2_ with and without NaF addition. Reaction conditions: [CIP] = 10 mg·L^−1^, [Bi_12_FeO_20_] = 0.5 g·L^−1^, [H_2_O_2_] = 2 mm, [IPA] = 5 mm, [NaBrO_3_] = [KI] = 1 mm, [PDA] = 10 ppm.

To elucidate the underlying mechanism responsible for the high mineralization capacity of the toxic quinone‐imine (P9) intermediate in the Bi_12_FeO_20_‐H_2_O_2_ system, we first investigate the role of ROS by adding different capture reagents. Initially, CIP undergoes degradation in the H_2_O_2_‐alone system for 8 h to ensure complete conversion to quinone‐imine intermediates. Subsequently, under an N_2_ atmosphere, specific capture reagents are introduced to capture different ROS species: polydopamine (PDA) nanoparticles for ·O_2_^−^, IPA for ·OH, sodium bromate (NaBrO_3_) for e^−^ [39], and potassium iodide (KI) for h^+^ [40], as shown in Figure 6a and Figure S22. The variations in the P9 peak area are monitored in the presence of these capture reagents to assess their impact on the mineralization process.

As a control experiment, in the absence of any scavengers, the quinone‐imine intermediates (P9) in the Bi_12_FeO_20_‐H_2_O_2_ system are degraded by 86.2% within 150 min, corresponding to a degradation rate constant of 0.011 min^−1^. Upon scavenging ·O_2_^−^ and e^−^, the degradation of the P9 intermediate decreased to 49.7% and 47.6%, respectively. In comparison, scavenging ·OH and h^+^ resulted in P9 degradation rates of 76.1% and 62.7%, respectively, indicating a relatively smaller impact on quinone‐imine mineralization. Given that e^−^ is a prerequisite for ·O_2_^−^ generation, and that ·O_2_^−^ and e^−^ exhibit nearly identical effects on P9 degradation, it can be inferred that the removal of quinone‐imine intermediates primarily depends on ·O_2_^−^, with e^−^ contributing indirectly through its conversion to ·O_2_^−^.

Figure 6b compares EPR results after 5 min of irradiation in water and methanol within the Bi_12_FeO_20_‐H_2_O_2_ system, using DMPO as the radical capture reagent. Methanol typically detects ·O_2_^−^, while water detects ·OH due to DMPO's higher affinity for water, usually resulting in negligible DMPO‐O_2_^−^· signals in water [19]. However, in the Bi_12_FeO_20_‐H_2_O_2_ system, both DMPO‐OH· and DMPO‐O_2_^−^· signals are observed in water, unlike the H_2_O_2_‐alone system, where only DMPO‐OH· is detected(Figure S12). This indicates that ·O_2_^−^ may exist on the surface of the Bi_12_FeO_20_ catalyst, showcasing a unique feature of this system. Subsequently, the addition of CIP results in the disappearance of the DMPO‐O_2_^−^· signal in water, with only the DMPO‐OH· signal remaining, which demonstrates that ·O_2_^−^ plays a crucial role in the CIP degradation process under these conditions.

To further investigate the reaction mechanism on the catalyst surface, we employ in situ FTIR to monitor the surface‐bound ·O_2_^−^ in real time. As shown in Figure 6c, with the extension of irradiation time, characteristic absorption peaks appear at 1048, 1222, and 1400 cm^−1^, which are attributed to the characteristic stretching vibrations of key intermediate species, including superoxide radicals (^*^O_2_^−^), hydroperoxyl radicals (^*^OO‐H), and hydrogen peroxide (^*^HOOH) [41, 42]. This result not only confirms the activation process of H_2_O_2_ on the catalyst surface but also reveals the activation pathway with ·O_2_^−^ as the main product, providing direct evidence for the existence of surface‐bound ·O_2_^−^.

To quantify ·O_2_^−^ under different statuses, sodium fluoride (NaF) is introduced to differentiate between free and surface‐bound ·O_2_^−^. F^−^ ions form strong coordination bonds on the oxide surface, releasing surface‐bound ·O_2_^−^ into the solution [43]. The concentration of ·O_2_^−^ in the Bi_12_FeO_20_‐alone and Bi_12_FeO_20_‐H_2_O_2_ systems, with and without NaF addition, is quantified using nitroblue tetrazolium (NBT) (Figure 6d; Text S6 and Figure S23). In the Bi_12_FeO_20_‐alone system, a small amount of ·O_2_^−^ (1.998 nm) is detected due to dissolved oxygen, and NaF addition does not significantly increase its concentration (1.842 nm), indicating no contribution to ·O_2_^−^ generation. In contrast, in the Bi_12_FeO_20_‐H_2_O_2_ system, NaF addition increases the ·O_2_^−^ concentration by 24.7%, from 6.191 to 8.226 nm, confirming the release of surface‐bound ·O_2_^−^. In addition, nitrobenzene (NB) is employed to determine the steady‐state concentration of ·OH, demonstrating that only trace amounts of free ·OH and surface‐bound ·OH are present in the Bi_12_FeO_20_‐H_2_O_2_ system compared with ·O_2_^−^ (Figure S24).

EPR analysis confirms the presence of surface‐bound ·O_2_^−^, with the DMPO‐O_2_^−^· signal intensity increasing by 46.5% upon NaF addition, indicating its release into the solution (Figure S25). Combined with capture experiments, in situ FTIR, NBT quantification, and EPR results, surface‐bound ·O_2_^−^ is identified as the dominant active radical, likely driving quinone‐imine intermediate degradation. For comparison, pyrite (FeS_2_), a heterogeneous Fenton catalyst, exhibits negligible surface‐bound ·O_2_^−^ and demonstrates no degradation of quinone‐imine intermediates after 150 min of reaction, as shown in Figure S26.

**FIGURE 7:**
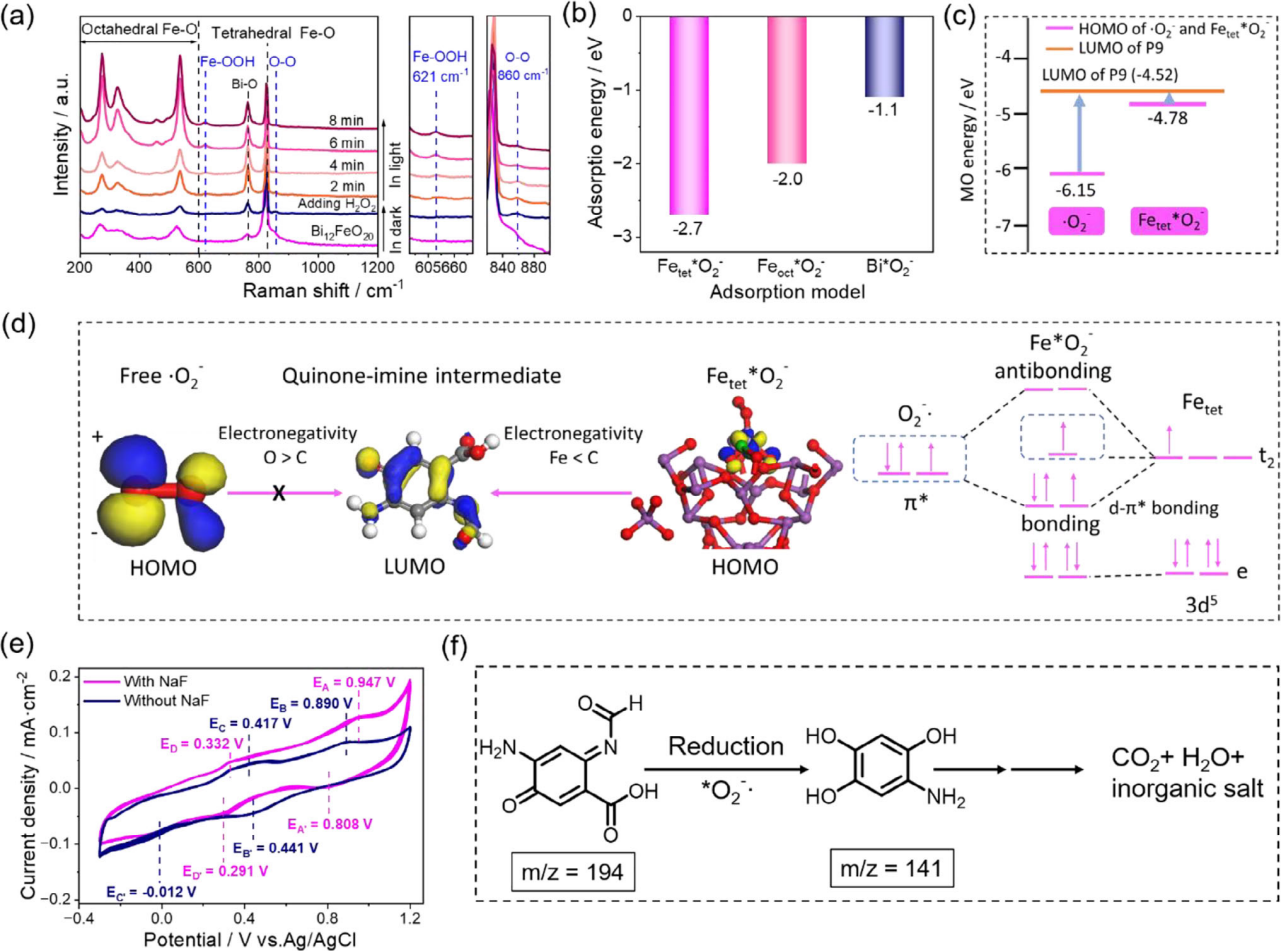
(a) In situ Raman spectra of the Bi_12_FeO_20_‐H_2_O_2_ system recorded as a function of irradiation time and the magnifications. (b) Comparison of different adsorption energies of the Bi_12_FeO_20_ catalyst. (c) Calculated energy levels of the LUMO of P9 and the HOMO of free and adsorbed ·O_2_^−^. (d) Frontier molecular orbital map of free ·O_2_^−^, and surface‐bound ·O_2_^−^ and quinone‐imine intermediates. White, red, purple, gray, and blue represent H, O, Bi, C, and Fe atoms, respectively. (e) CV curves of H_2_O_2_ activation on the Bi_12_FeO_20_ electrode with and without 50 mM NaF. (f) Schematic diagram of the degradation pathway of quinone‐imine intermediates under the surface‐bound ·O_2_^−^.

To further clarify the role of surface‐bound ·O_2_^−^ during the reaction, in situ Raman spectroscopy is conducted in the Bi_12_FeO_20_‐H_2_O_2_ system under 523 nm laser excitation. As shown in Figure 7a, upon the addition of H_2_O_2_, a Raman peak emerges at 860 cm^−1^, corresponding to the O─O stretching vibration of ^*^OOH [44]. Upon exposure to light, a distinct Raman peak appears at 621 cm^−1^, which is assigned to the Fe─O stretching vibration of the ^*^OOH species absorbed onto the tetrahedral Fe atoms [45, 46]. With prolonged irradiation, the intensities of the Fe─O Raman peaks consistently increase. This observation demonstrates that the tetrahedral Fe sites within the Bi_12_FeO_20_ catalyst are activated, facilitating the stepwise formation of Fe‐^*^OOH intermediates.

We then calculate the adsorption energies of ·O_2_^−^ on tetrahedral Fe, octahedral Fe, and octahedral Bi atoms (Figure 7b; Figure S27). The calculated results reveal a significantly stronger adsorption affinity of ·O_2_^−^ on tetrahedral Fe atoms, with an adsorption energy of −2.7 eV, which is more negative than that for octahedral Fe atoms (−2.0 eV) and for Bi atoms (−1.1 eV). This significant energy difference demonstrates that ·O_2_^−^ preferentially binds to tetrahedral Fe atoms within the catalyst structure, consistent with the in situ Raman observations. Further investigation into the degradation of quinone‐imine intermediates through H_2_O_2_ activation by Bi_2_O_3_ nanosheets revealed a limited degradation efficiency of only 32.7% (Figure S28). This result underscores the critical role of the adsorption of ·O_2_^−^ onto tetrahedral Fe sites in facilitating the effective degradation.

Figure 7c presents a comparative analysis of the calculated energy levels between the LUMO of the quinone‐imine intermediate and the HOMO of free ·O_2_^−^ and surface‐bound ·O_2_^−^. This energy level comparison provides crucial insights into the charge transfer feasibility between these frontier orbitals, thereby elucidating the relative reduction capabilities of free and surface‐bound ·O_2_^−^ toward the quinone‐imine intermediate [47]. The analysis reveals that the HOMO of tetrahedral Fe^*^O_2_^−^ (−4.78 eV) is slightly lower in energy compared to that of the LUMO of quinone‐imine intermediate (−4.52 eV), suggesting a favorable electron transfer pathway. In contrast, the HOMO energy level of free ·O_2_^−^ (−6.15 eV) is substantially lower than that of the quinone‐imine intermediate, indicating a less favorable electron transfer process. This distinct energy level alignment demonstrates that the quinone‐imine intermediate preferentially accepts electrons from the HOMO of the surface‐bound ·O_2_^−^ rather than from free ·O_2_^−^, highlighting the enhanced reactivity of the surface‐bound species in the reduction process.

The map of the frontier molecular orbital is further calculated to understand the electron transfer from the HOMO of ·O_2_^−^ to the LUMO of the quinone‐imine intermediate, as shown in Figure 7d. For free ·O_2_^−^, to achieve such transfer, an electron needs to transfer from the π^*^ antibonding orbitals of O to the C orbitals on the benzene ring. However, the electronegativity of O (3.44) is greater than that of C (2.55), making it difficult for such a transfer. For surface‐bound ·O_2_^−^ on tetrahedral Fe atoms, the situation is changed. First, the origin‐filled π^*^ antibonding orbitals of ·O_2_^−^ will form a new bonding orbital with the empty t_2_ orbitals of Fe atoms by the d‐π^*^ bonding, exhibiting an energy level lower than the originally HOMO in free ·O_2_^−^ as suggested by the calculated PDOS of Fe and O, and the COHP of Fe─O bonding (Figure S29). Second, the LUMO orbitals will change from the π^*^ antibonding orbitals of free ·O_2_^−^ to the filled t_2_ orbitals of the tetrahedrally‐coordinated Fe atoms. Since the electronegativity of Fe (1.83) is smaller than that of C (2.55), the electron transfer from the newly formed HOMO of surface‐bound ·O_2_^−^ will be easier relative to free ·O_2_^−^, facilitating the reduction of the quinone‐imine intermediate and promoting the next degradation.

Next, we employ cyclic voltammetry (CV) to investigate the change of redox behavior of free and adsorbed ·O_2_^−^ in a 0.5 m Na_2_SO_4_ electrolyte containing 0.1 mm H_2_O_2_. NaF is added to weaken the adsorption of ·O_2_^−^ onto the Bi_12_FeO_20_ due to the specific adsorption of F^−^ ions on the catalyst surface, and an Ag/AgCl electrode (0.210 V vs. NHE) is used as a reference. As illustrated in Figure 7e, without NaF where surface‐bound ·O_2_^−^ predominantly exist, two distinct redox peaks appear at 0.417/−0.012 V and 0.890/0.441 V, corresponding to the conversions of O2/·O_2_^−^ and ·O_2_^−^/H_2_O_2_, respectively [48, 49]. Accordingly, the reduction potential for ·O_2_^−^/O_2_ calculates to be 0.203 V. Upon introducing 50 mm NaF, where the strong adsorption of ·O_2_^−^ is weakened, two similar redox peaks appear at 0.332/0.291 V and 0.947/0.808 V, and thus the reduction potential of 0.312 V for O_2_/·O_2_^−^. The reduction potential of the free‐state ·O_2_^−^ is more positive than that of the surface‐bound ·O_2_^−^, indicating that the surface‐bound ·O_2_^−^ exhibits significantly enhanced reducing capability.

The gas chromatography‐mass spectrometry (GC‐MS) analysis is employed to reveal the degradation of the quinone‐imine intermediate. The result shows that the quinone‐imine intermediate (P9, m/z = 194) undergoes a reduction process facilitated by surface‐bound ·O_2_^−^ as indicated by the DFT calculations and electrochemical tests, leading to the formation of polyhydroxy phenols (m/z = 141) (Figure 7f; Figure S30). This experimental observation confirms our theoretical prediction that surface‐bound ·O_2_^−^ exhibits stronger reducing capability than its free‐state counterpart. The reduced polyhydroxy phenols are subsequently oxidized to form quinone compounds, which then undergo benzene ring opening followed by carboxylation. This sequential reaction pathway ultimately results in complete mineralization, producing CO_2_, H_2_O, and inorganic salts as the final products (Figure S31), which is consistent with previous reports [50, 51]. These findings demonstrate that surface‐bound ·O_2_^−^ plays a crucial role in initiating the degradation pathway of the quinone‐imine intermediate through its enhanced reducing capacity, as predicted by our theoretical calculations.

### 2.4 The Removal of CIP and Intermediates in an Actual Water System with the Bi_12_FeO_20_‐H_2_O_2_ System

**FIGURE 8:**
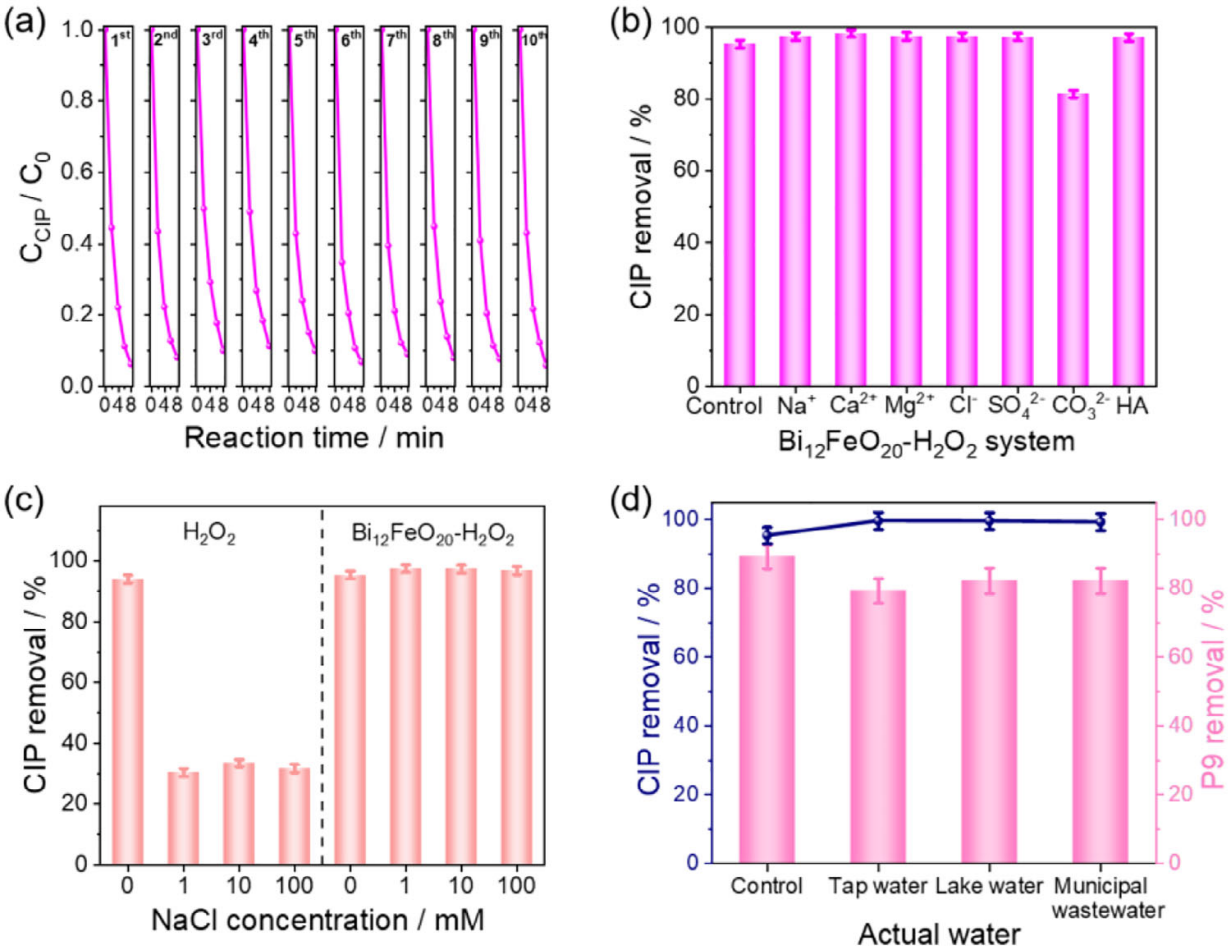
(a) The cyclic experiment of CIP degradation in the Bi_12_FeO_20_‐H_2_O_2_ system. (b) The CIP removal rate in the presence of various ions and HA in the Bi_12_FeO_20_‐H_2_O_2_ system. (c) The CIP removal rate in different NaCl (1, 10, and 100 mm) concentrations in the Bi_12_FeO_20_‐H_2_O_2_ and H_2_O_2_‐alone systems. (d) The CIP and quinone‐imine intermediates (P9) removal percentage in the Bi_12_FeO_20_‐H_2_O_2_ system in various water bodies (tap water, lake water, and municipal wastewater). Degradation conditions: [CIP] = 10 mg·L^−1^, [Bi_12_FeO_20_] = 0.5 g·L^−1^, [H_2_O_2_] = 2 mm, [Na^+^] = [Ca^2+^] = [Mg^2+^] = [Cl^−^] = [SO_4_^2−^] = [CO_3_^2−^] = 1 mm, [HA] = 5 ppm, the quinone‐imine intermediates are obtained by the CIP solution in the H_2_O_2_‐alone system reacting for 8 h.

To assess the potential application of the Bi_12_FeO_20_‐H_2_O_2_ Photo‐Fenton system, its durability is systematically evaluated. The CIP degradation efficiency in the Bi_12_FeO_20_‐H_2_O_2_ system demonstrates remarkable stability over 10 cycles in Figure 8a, with a minimal standard deviation of 0.02008. In addition, we examine the recovery rate of the catalyst before and after the reaction, and the results indicate a recovery rate of 99.6%, thereby confirming the system's excellent durability for CIP removal. Furthermore, XRD analysis (Figure S32) reveals no structural alterations in Bi_12_FeO_20_ before and after the reaction, indicating the significant structural stability of the Fe‐sillenite material. TEM images of the Bi_12_FeO_20_ catalyst after the reaction (Figure S33) show that the nanosheet morphology remains unchanged before and after the reaction. Quantitative analysis by ICP‐OES (Table S6) shows that the Bi and Fe leakage from Bi_12_FeO_20_ into the solution is only 0.014% and 0.041% after 100 min of Photo‐Fenton reaction. These values are significantly lower than the reported iron leakage rates of 0.37% for FeS_2_ and 0.1% for Fe_2_O_3_ salts [52, 53], respectively, highlighting the superior stability and reduced metal leaching of the Bi_12_FeO_20_ catalyst in the Fenton‐like process.

Figure 8b illustrates the degradation efficiency of CIP in the Bi_12_FeO_20_‐H_2_O_2_ system under the influence of various ions and humic acid (HA). The system demonstrates robust performance with CIP removal rates of 97.5%, 98.3%, 97.5%, 97.5%, 97.3%, 81.4%, and 97.2% in the presence of Na^+^, Ca^2+^, Mg^2+^, Cl^−^, SO_4_^2−^, CO_3_^2−^, and HA, respectively. These findings underscore the Bi_12_FeO_20_‐H_2_O_2_ system to effectively mitigate the interference from diverse ionic species and HA. Moreover, the system demonstrates significant efficacy in promoting the removal of CIP across various environmental conditions.

Figure 8c illustrates the degradation efficiency of CIP in the Bi_12_FeO_20_‐H_2_O_2_ and H_2_O_2_‐alone system across varying sodium chloride (NaCl) concentrations (1–100 mm). In the H_2_O_2_‐alone system, the presence of NaCl results in a limited CIP degradation efficiency of only 32% within 8 min. In contrast, the Bi_12_FeO_20_‐H_2_O_2_ system demonstrates significantly enhanced performance, achieving an average CIP degradation efficiency of 97.2%. The absence of a Cl 2p peak in wide‐scan XPS confirms negligible Cl^−^ adsorption (Figure S34), enabling surface‐bound ·O_2_^−^ in Bi_12_FeO_20_‐H_2_O_2_ to counteract Cl^−^ quenching and degrade CIP efficiently [54, 55].

Figure 8d illustrates the remarkable removal efficiency of CIP and its quinone‐imine intermediates (P9) by the Bi_12_FeO_20_‐H_2_O_2_ system in various actual waters. The Bi_12_FeO_20_‐H_2_O_2_ system exhibited remarkable performance in CIP degradation, achieving removal efficiencies of 99.6%, 99.5%, and 99.2% within 8 min in tap water, lake water, and municipal wastewater matrices, respectively. Notably, the system demonstrated significant capability in eliminating quinone‐imine intermediates, with removal efficiencies of 79.3%, 82.2%, and 82.1% after 200 min in the respective water samples, compared to 89.2% in deionized water controls. In addition, we further apply the Bi_12_FeO_20_‐H_2_O_2_ system to degrade CIP in actual pharmaceutical wastewater, achieving a remarkable CIP degradation efficiency of 99.4% within 30 min (Figure S35). The actual pharmaceutical wastewater contains a large amount of alcohols and pharmaceutical active ingredients with aromatic ring structures, along with gel‐sol impurities. These complex coexisting organic components competitively react with ROS in the system, thereby reducing the effective oxidation efficiency and consequently prolonging the time required for the degradation process. These findings underscore the system's dual functionality in water treatment: not only does it efficiently degrade CIP, but it also effectively removes the potentially toxic intermediates generated during the degradation process. The consistently high performance of this system across various real water matrices demonstrates its robustness and provides a feasible approach for the treatment of complex actual water systems.

## 3 Conclusion

This study develops an iron‐based sillenite (Bi_12_FeO_20_) nanosheet system for H_2_O_2_ activation and reveals its interfacial radical‐regulation capability for deep detoxification of antibiotics. The Bi_12_FeO_20_‐H_2_O_2_ photo‐Fenton system achieves rapid degradation of CIP (94.3% in 8 min) and deep mineralization (TOC 93.8%), markedly outperforming conventional Fenton systems. Mechanistic investigations show that the Fe─O tetrahedral sites stably generate surface‐bound ·O_2_^−^ and raise its HOMO energy level, thereby enhancing its interfacial reducing power. The surface‐bound ·O_2_^−^ efficiently injects electrons into the LUMO of the quinone‐imine intermediate, initiating ring‐opening reduction followed by oxidation‐mineralization reactions and ultimately destroying the refractory structure. This surface‐bound radical‐driven interfacial reduction–oxidation coupled mechanism overcomes the inherent limitations of free ROS in eliminating persistent intermediates. Overall, this work uncovers the structural advantages of sillenite in advanced oxidation processes and provides a new strategy for tailoring the electronic structure and interfacial radical chemistry of Fenton catalysts, demonstrating substantial potential for practical antibiotic remediation in water environments.

## 4 Experimental Section

### 4.1 Chemicals and Reagents

Bismuth nitrate pentahydrate (Bi(NO_3_)_3_·5H_2_O) (AR, purity ≥ 99%), sodium hydroxide (NaOH) (≥97%), Iron(III) chloride hexahydrate (FeCl_3_·6H_2_O) (AR, ≥ 99%), Chromium(III) nitrate nonahydrate (Cr(NO_3_)_3_·9H_2_O), methanol (CH_3_OH) (HPLC), isopropyl alcohol (IPA) (70%), silver nitrate (AgNO_3_) (AR), potassium iodide (KI) (99.5%) and p‐benzoquinone (p‐BQ) (99%) are purchased from the National Chemical Company (Shanghai, China) in analytical grade. Ciprofloxacin (CIP) (≥98%), ofloxacin (OFX) (≥98%), and norfloxacin (NFX) (≥98%) are purchased from J&K Scientific (Beijing, China) in analytical grade and used directly without further purification. Water with a resistance of 18.2 MΩ (Ultrapure water) is employed for all experiments. The tap water comes from the laboratory of Jiangnan University in Wuxi, China; the lake water source used in the experiment is sourced from Taihu Lake (Longitude: 120.2°, Latitude: 31.48°); and the municipal wastewater is collected after secondary treatment (activated sludge and secondary clarifier) at a municipal wastewater treatment plant in Wuxi, China.

### 4.2 Material Characterization

X‐ray diffraction (XRD) patterns are recorded using a Bruker D8‐ADVANCE diffractometer with Cu Kα1 radiation (λ = 1.5406 Å), operating at 40 kV and 40 mA. The morphology of the samples is examined using scanning electron microscopy (SEM) with a Hitachi S‐4800, while transmission electron microscopy (TEM) is conducted with a JEOL JEM‐2100plus. In situ Raman spectroscopy measurements are performed using a Smart Raman confocal micro‐Raman module equipped with a 50× objective lens, employing a backscattering geometry. This module, developed by the Institute of Semiconductors of the Chinese Academy of Sciences, is paired with a Horiba iHR550 spectrometer, employing a 532 nm excitation laser and a charge‐coupled device (CCD) detector for data acquisition. Catalyst thickness measurements are carried out with an atomic force microscope (AFM, SPM‐9700HT, Shimadzu). For X‐ray photoelectron spectroscopy (XPS), an AXIS Supra instrument (Kratos UK) is utilized, employing monochromatic Al Kα radiation (hv = 1486.6 eV, 225 W), with calibration against the C1s peak (284.8 eV). Hydroxyl radicals and superoxide radicals are assessed under both dark and illuminated conditions using a 300W Xe lamp for 5 min, with an electron paramagnetic resonance (EPR, Bruker EMXplus, Germany) spectrometer. UV–vis diffuse reflectance spectra are acquired using a UV‐3600 spectrophotometer (Shimadzu, Japan).

### 4.3 Preparation of Bi_12_FeO_20_ ((Bi^III^_23_Fe^III^)(Bi^V^Fe^III^O_8_)O_32_) Sillenite Nanosheets

Bi_12_FeO_20_ nanosheets are produced via a hydrothermal method as detailed in our previous study [23]. In this process, Bi(NO_3_)_3_·5H_2_O (0.582g, 1.2 mmol), FeCl_3_·6H_2_O (0.027g, 0.1 mmol), and Cr(NO_3_)_3_·9H_2_O (0.040g, 0.1 mmol) are added to NaOH solution (2.400g, 2 mol·L^−1^) to create a yellow‐green suspension. This mixture is stirred at 660 rpm for 30 min before being placed in a 50 mL stainless steel autoclave sealed with para‐polyphenol. The autoclave is maintained at 180°C for 12 h and then cooled to room temperature, yielding a dark, reddish‐brown precipitate and a light green solution that contains [CrO_4_]^−^ cations (soft templates during the nanosheet preparation). The precipitate is collected by centrifugation and rinsed three times with ultrapure water (18 MΩ cm) until the pH reaches 7. Finally, the product is dried overnight at 60°C to yield Bi_12_FeO_20_ nanosheets.

### 4.4 Photo‐Fenton Degradation Performance

The Photo‐Fenton performance of the prepared Bi_12_FeO_20_ is assessed by degrading CIP. Bi_12_FeO_20_ (0.025g, 0.5 g·L^−1^) is mixed with a CIP aqueous solution (10 ppm, 50 mL) and subjected to ultrasonication for 5 min to create a uniform suspension. Before the degradation process, the mixture is stirred in the dark for 30 min to achieve adsorption equilibrium. Next, H_2_O_2_ (2 mM) is added to the suspension, and an Xe lamp (300 W, λ > 300 nm, Perfect Light, Beijing, China) is employed to initiate the degradation process. Every 2 min, 1 mL of the reaction solution is withdrawn using a syringe equipped with a 0.22 µm filter to separate the catalyst. The concentration of CIP and its degradation intermediates is analyzed via high‐performance liquid chromatography (HPLC, Ultimate 3000RS, JASCO, Japan) with a C18 reverse phase column, utilizing a mobile phase flow rate (V_methanol_: V_formic acid aq (0.1 vol%)_ = 3.5: 6.5) of 1.0 mL·min^−1^. The detection wavelength is maintained at 272 nm.

### 4.5 Scavenging Experiment

The specific scavengers are employed to selectively capture different types of radicals: isopropanol (IPA) for hydroxyl radicals (∙OH), 1,4‐benzoquinone (p‐BQ)/ polydopamine particles (PDA) for superoxide radicals (∙O_2_^−^), silver nitrate (AgNO_3_)/ sodium bromate (NaBrO_3_) for electrons (e^−^), potassium iodide (KI) for holes (h^+^), and L‐histidine (L‐His) for singlet oxygen (^1^O_2_). The presence of reactive oxygen species (ROS) is detected using electron paramagnetic resonance (EPR) spectroscopy and DMPO as a spin‐trapping agent, while nitroblue tetrazolium (NBT) is utilized as a probe to quantitatively measure the concentration of superoxide radicals (∙O_2_^−^). In the capture experiment for quinone‐imine intermediates, a 10 ppm CIP solution is first mixed with 2 mM H_2_O_2_ and irradiated under a 300 W xenon lamp for 8 h to generate the initial degradation solution (C_0_). Subsequently, the Bi_12_FeO_20_ catalyst (25 mg) and a specific concentration of the scavenger are added to the degradation solution. The degradation of quinone‐imine intermediates is analyzed using HPLC to verify the scavenging effect. Additionally, sodium fluoride (NaF) is added to detect surface‐bound ∙O_2_^−^, and the degradation of NBT is monitored using UV–vis spectroscopy to further analyze the generation and reaction mechanisms of the radicals. Detailed experimental procedures and conditions are provided in Text S6.

### 4.6 Photoelectrochemical Measurements

Photoelectrochemical measurements are conducted using a CHI 750E electrochemical workstation (Chenhua, Shanghai, China). The system employs a three‐electrode configuration, with the photocatalyst/FTO glass (1 cm × 1 cm) serving as the working electrode. A platinum net (1.5 cm × 1.5 cm) acts as the counter electrode, and a standard Ag/AgCl electrode (E = 0.210 V vs. NHE) functions as the reference electrode. The electrolyte consists of a 0.5 mol/L sodium sulfate solution (150 mL). A 300 W Xenon lamp provides the light source. To prepare the photocatalyst/FTO working electrode, the drop‐casting method is applied. Specifically, 2 mg of the photocatalyst is dispersed in 500 µL of ultrapure water through 5 min of ultrasonication. The resulting suspension is evenly dropped onto an FTO conductive glass and allowed to air‐dry, forming the electrode.

### 4.7 Theoretical Calculation

The adsorption energy as well as the LUMO and HOMO levels of free ·O_2_^−^, Bi_12_FeO_20_ surface‐bound ·O_2_^−^, and CIP are calculated using Material Studio with the Dmol^3^ package [55]. The exchange and correlation functional is treated with the generalized gradient approximation (GGA–PW91), taking into account the bulk solvent effects of water. A Bi_12_FeO_20_ slab model is constructed based on the (200) planes with a thickness of two layers, consistent with TEM observations, and separated by a vacuum gap of 15 Å. For a better description of the d orbitals of transition metals, we also used a Hubbard U parameter for the 3d orbital of low‐spin Fe, which was set to 2.5 eV. The tolerances for geometry optimization are set with the total energy of 1 × 10^−4^ eV/atom, the maximum force of 0.02 eV/Å, and the maximum ionic displacement of 0.05 Å. The adsorption energy is calculated as E_ads_ = E(Bi_12_FeO_20_^*^O_2_^−^)‐E(Bi_12_FeO_20_)‐E(free ·O_2_^−^).

## Conflicts of Interest

The authors declare no conflict of interest.

## Supporting information




**Supporting File**: advs74822‐sup‐0001‐SuppMat.docx.

## Data Availability

The data that support the findings of this study are available from the corresponding author upon reasonable request.
