# Supplementary material for: Surface‐Bound Superoxide Radical‐Mediated Photo‐Fenton Mineralization of Ciprofloxacin on Fe‐Sillenite Nanosheets
Source: Adv Sci (Weinh). 2026 Mar 16;13(29):e22479. doi: 10.1002/advs.202522479 (PMC13205808; doi:10.1002/advs.202522479)
Supplement: Supplementary file 1 — Supporting File: advs74822‐sup‐0001‐SuppMat.docx. [file ADVS-13-e22479-s001.docx]

Supporting Information

**Surface-Bound Superoxide Radical-Mediated Photo-Fenton Mineralization of Ciprofloxacin on Fe-Sillenite Nanosheets**

Wenting Qiu, Ji Liu, Hengjun Shang, Yaning Zhang, Shuai Dou, Jing Xu, Ying Zhang, Yang Lou, Yongfa Zhu, and Chengsi Pan*

Wenting Qiu, Ji Liu, Hengjun Shang, Yaning Zhang, Shuai Dou, Ying Zhang, Yang Lou, Chengsi Pan

International Joint Research Center for Photoresponsive Molecules and Materials

Jiangnan University

Wuxi, Jiangsu, 214122, China

E-mail: [cspan@jiangnan.edu.cn](mailto:cspan@jiangnan.edu.cn%20) (C. Pan)

Wenting Qiu, Ji Liu, Hengjun Shang, Yaning Zhang, Shuai Dou, Ying Zhang, Yang Lou, Chengsi Pan

School of Chemical and Materials Engineering

Jiangnan University

Wuxi, Jiangsu, 214122, China

Jing Xu

School of Food Science and Technology

Jiangnan University

Wuxi, Jiangsu, 214122, China

Yongfa Zhu

Department of Chemistry

Tsinghua University

Beijing, 100086, China

**Content**

**Text S1.** The reaction kinetics.

**Text S2.** Bandgap calculation.

**Text S3.** Determination of H_2_O_2_ concentration.

**Text S4.** Calculation of H_2_O_2_ utilization efficiency.

**Text S5.** Toxicity experiments.

**Text S6.** Identification of ·O_2_^-^.

**Figure S1.** XRD of prepared Bi_12_FeO_20_ nanosheets.

**Table S1.** XRD refinement parameters for Bi_12_FeO_20_ nanosheets.

**Figure S2.** SEM-EDX of prepared Bi_12_FeO_20_ nanosheets.

**Figure S3.** Thermogravimetric analysis of the Bi_12_FeO_20_ nanosheet catalyst.

**Figure S4.** Mott-Schottky curve of the prepared Bi_12_FeO_20_ nanosheets.

**Figure S5.** The degradation curve of CIP under dark conditions and CIP photodegradation.

**Figure S6.** Isothermal adsorption-desorption curves of prepared Bi_12_FeO_20_ nanosheets.

**Figure S7.** The optimization of the CIP degradation process under different conditions.

**Figure S8.** Photo-Fenton degradation performance of different CIP concentrations (1 ppm and 5 ppm) in the Bi_12_FeO_20_-H_2_O_2_ system.

**Figure S9.** The Bi_12_FeO_20_ catalyst activates PMS to degrade CIP.

**Figure S10.** The decomposition rate of H_2_O_2_ in the Bi_12_FeO_20_-H_2_O_2_ and H_2_O_2_-alone systems.

**Table S2.** The utilization efficiency of H_2_O_2_ in the Bi_12_FeO_20_-H_2_O_2_ and H_2_O_2_-alone systems.

**Figure S11.** Capture experiments in the H_2_O_2_-alone and Bi_12_FeO_20_-H_2_O_2_ systems.

**Figure S12.** EPR spectra of the H_2_O_2_-alone and Bi_12_FeO_20_-H_2_O_2_ systems.

**Figure S13.** The degradation of CIP in the Bi_12_FeO_20_-H_2_O_2_ system in Ar and air.

**Figure S14.** RRDE test of the Bi_12_FeO_20_-H_2_O_2_ system.

**Figure S15.** Comparison of the other Fe-Fenton systems.

**Figure S16.** Comparison of the changes in peak areas of the intermediates.

**Figure S17.** The HPLC spectra of P9 degradation in the H_2_O_2_-alone system.

**Figure S18.** The LC-MS spectra of the CIP and the intermediates produced by CIP degradation.

**Table S3.** The CIP and its intermediates are systematically analyzed and identified.

**Figure S19.** HPLC spectra of other fluoroquinolones degradation in the H₂O₂-alone system.

**Table S4.** The toxicity assessment levels.

**Figure S20.** The T.E.S.T. software performs the toxicity prediction.

**Figure S21.** The colony images and the relative bacterial viability of the toxicity test.

**Table S5.** The growth patterns of soybean sprouts under various conditions.

**Figure S22.**The degradation rate constant of the P9 intermediates in the Bi_12_FeO_20_-H_2_O_2_ system.

**Figure S23.** The NBT consumption rate in the Bi_12_FeO_20_-alone and Bi_12_FeO_20_-H_2_O_2_ systems.

**Figure S24.** Comparison of the ·OH concentration in the H_2_O_2_-alone and Bi_12_FeO_20_-H_2_O_2_ with and without NaF addition.

**Figure S25.** EPR spectra of the DMPO-O_2_^-^· signals in the Bi_12_FeO_20_-H_2_O_2_ system.

**Figure S26.** The P9 intermediate degradation rate by pyrite (FeS_2_) H_2_O_2_-Fenton system corresponds to the NBT consumption with and without NaF addition.

**Figure S27.** The optimized structure models on the (200) surface of Bi_12_FeO_20_. And corresponding to adsorption structure models of Bi*O_2_^-^, Fe_tet_*O_2_^-,^ and Fe_oct_*O_2_^-^.

**Figure S28.** Degradation of P9 in the Bi_2_O_3_-H_2_O_2_ system.

**Figure S29.** The partial density of states (PDOS) calculations for Bi_12_FeO_20_.

**Figure S30.** The GC-MS spectrum and LC-MS spectrum of the m/z=141 intermediate.

**Figure S31.** Possible intermediates of polyhydroxy phenols in the Bi_12_FeO_20_-H_2_O_2_ system and the GC-MS spectra of the m/z=123, 135, and 60 intermediates.

**Figure S32.** XRD of fresh and used reaction at prepared Bi_12_FeO_20_ nanosheets.

**Figure S33.** TEM images of Bi_12_FeO_20_ after repeated reaction cycles.

**Table S6.** ICP-OES results of the Fe-leakage of Bi_12_FeO_20_ into the different solutions.

**Figure S34.** The XPS spectra before and after NaCl soaking at the prepared Bi_12_FeO_20_ nanosheets.

**Figure S35.** Degradation efficiency of CIP at a concentration of 5 ppm in pharmaceutical wastewater using the Bi_12_FeO_20_-H_2_O_2_ system.

**Text S1. The reaction kinetics**

The experiments employ the pseudo-first-order kinetic model to analyze the degradation of CIP and P9 intermediates, described by the following equation:

$$\ln\left( \frac{C_{0}}{C_{t}} \right)= -k\cdot t (Equation S1)$$

The C_t_ is the concentration of CIP or P9 intermediates at a specific time, C_0_ is their initial concentration, k is the observed rate constant, and t is the reaction time.

**Text S2. Bandgap calculation**

The CBM and VBM energy levels of nanosheets are determined based on their flat band potentials. As outlined in the literature,^[1]^ the band positions are calculated using the equations:

$E_{CB}=E_{fb}+E_{(Ag/AgCl)} - E_{0}$(Equation S2)

$E_{VB}=E_{CB}+E_{g}$ (Equation S3)

Where E_fb_ is the flat band potential, E_CB_ and E_VB_ represent the conduction and valence band energies, respectively, and E_g_ is the bandgap energy. E_(Ag/AgCl)_ refers to the standard Ag/AgCl electrode (0.210 V vs. NHE), while E_0_ is the offset between the flat-band potential and the conduction band.

**Text S3. Determination of H_2_O_2_ concentration**

The H_2_O_2_ concentration in the reaction solution is determined using a potassium titanium oxide oxalate colorimetric method, measured with a UV-vis spectrophotometer (UV-3600, Shimadzu, Japan). In this process, 1.5 mL of the filtered sample is quickly and thoroughly mixed with an equal volume of 0.02 M potassium titanium oxalate solution. Following a two-minute color development period, the absorbance is measured at 400 nm using the spectrometer, enabling the analysis of the H_2_O_2_ concentration in the solution.^[2]^

**Text S4. Calculation of H_2_O_2_ utilization efficiency**

The H_2_O_2_ utilization efficiency (η(H_2_O_2_)) represents the ratio of the stoichiometric H_2_O_2_ consumption (Δ[H_2_O_2_]_s_) required for pollutant mineralization to the actual H_2_O_2_ consumption (Δ[H_2_O_2_]_a_) in the Fenton-like reaction, as expressed by the following equation:^[3]^

$$\eta(H_{2}O_{2}) = \Delta\left[ H_{2}O_{2} \right]_{s} / \Delta\left[ H_{2}O_{2} \right]_{a} (Equation S4)$$

Here, Δ[H_2_O_2_]_s_ is derived from the TOC concentration measurements before and after the reaction, while Δ[H_2_O_2_]_a_ is determined using a colorimetric method with potassium titanium oxide oxalate reagent at 400 nm (UV-3600, Shimadzu, Japan), as detailed in Text S3.

As shown in equation S5, the complete mineralization of one mole of CIP requires 20 moles of H_2_O_2_:

$C_{17}H_{18}O_{3}N_{3}F+ 20 H_{2}O_{2} \to17{CO}_{2}+54 H_{2}O+3{HNO}_{3}+$ HF (Equation S5)

**Text S5. Toxicity experiments**

The target strain selected for this study is Escherichia coli O157 (*E. coli O157*). The *E. coli O157* strains are obtained from a -80 °C freezer. After multiple purification steps, the bacterial suspension's optical density (OD) is adjusted to 1.0 at 600nm. The bacterial suspension is diluted to a concentration of 7.9 × 10⁷ colony-forming units per milliliter (CFU/mL) using sterile normal saline. Next, 1 mL of the bacterial suspension is combined with 9 mL of various solutions, including a 10 ppm CIP solution, the solution following H_2_O_2_-alone system degradation, and the solution after Bi_12_FeO_20_-H_2_O_2_ system degradation. The solution from the degradation of the H_2_O_2_-alone system is prepared by stirring and mixing 10 ppm CIP and 2 mM H_2_O_2_ for 2 h, followed by filtering. The solution from the degradation of the Bi_12_FeO_20_-H_2_O_2_ system is prepared by stirring and mixing 25 mg Bi_12_FeO_20_, 10 ppm CIP, and 2 mM H_2_O_2_ for 2 h, then filtering. All the degradation solutions are finally treated with 10 mg MnO_2_ to decompose the residual H_2_O_2_. These mixtures are then incubated for 12 h in a shaking incubator set at 37 °C. Following incubation, the bacterial suspension is serially diluted by a factor of 10³ and subsequently plated onto agar plates. After incubating the plates for 24 h at 37 °C, they are removed from the constant-temperature oven, and colony counts are performed using the plate count method. All experiments described above are repeated at least three times.

$Relative bacteria viability = {(C}_{t} / C_{0}$) × 100% (Equation S6)

C_0_ and C_t_ represent the number of colonies in normal saline and in the solutions treated with various systems, respectively.

**Text S6. Identification of** **·O_2_^-^**

Scheme 1 illustrates the interaction between superoxide ions and nitroblue tetrazolium (NBT), resulting in the formation of purple formazan. This compound is water-insoluble, causing it to precipitate on catalytic particles and impart a purple hue. The concentration of superoxide ions is quantified by tracking the reduction in NBT absorbance. In addition to the cumulative concentration, the steady-state concentration of ·O_2_^-^ is calculated using the following relationship:

$r_{int}=k_{\left( NBT+{\cdot O}_{2}^{-} \right)}\left[ NBT \right][{\cdot O}_{2}^{-}]$ (Equation S7)

Here, $r_{int}$ represents the initial degradation rate of NBT, $k_{(NBT+{\cdot O}_{2}^{-})}$ is the reaction rate constant between NBT and ·O_2_^-^ (reported as 5.88 × 10^4^ M^-1^·s^-1^), [NBT] is the initial NBT concentration (0.01 mM), and [·O_2_^-^] denotes the steady-state concentration of ·O_2_^-^. 3 mL of aqueous samples are taken at designated intervals, and the degradation of 0.01 mM NBT is analyzed at 259 nm using UV-Vis spectroscopy (UV-3600, Shimadzu, Japan). Spectra are measured from 200 to 500 nm, using deionized water as the reference.^[4]^


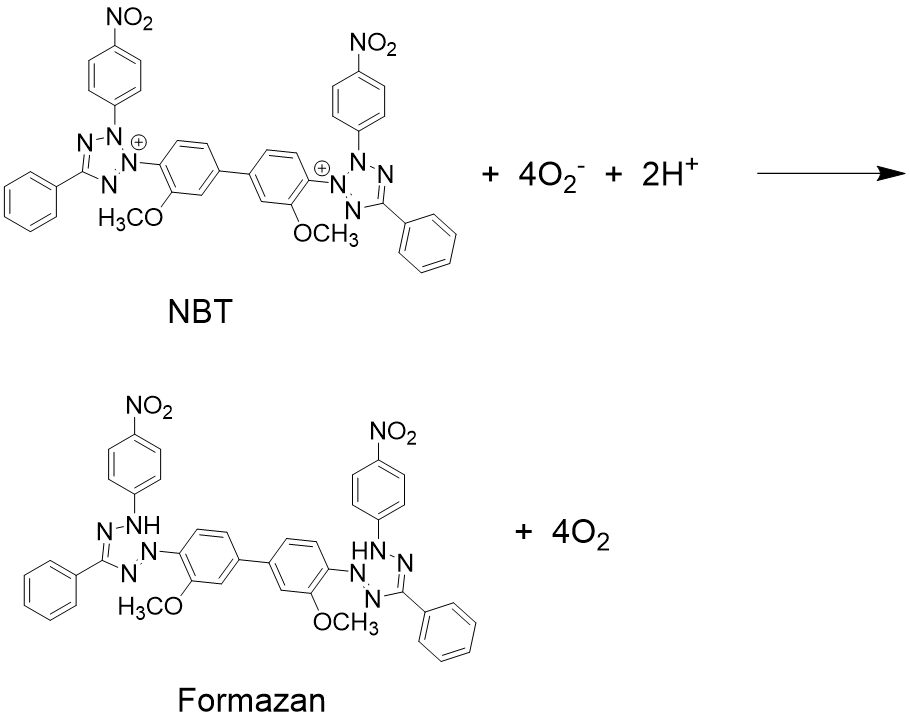


**Scheme 1.** NBT reacts with superoxide ions.


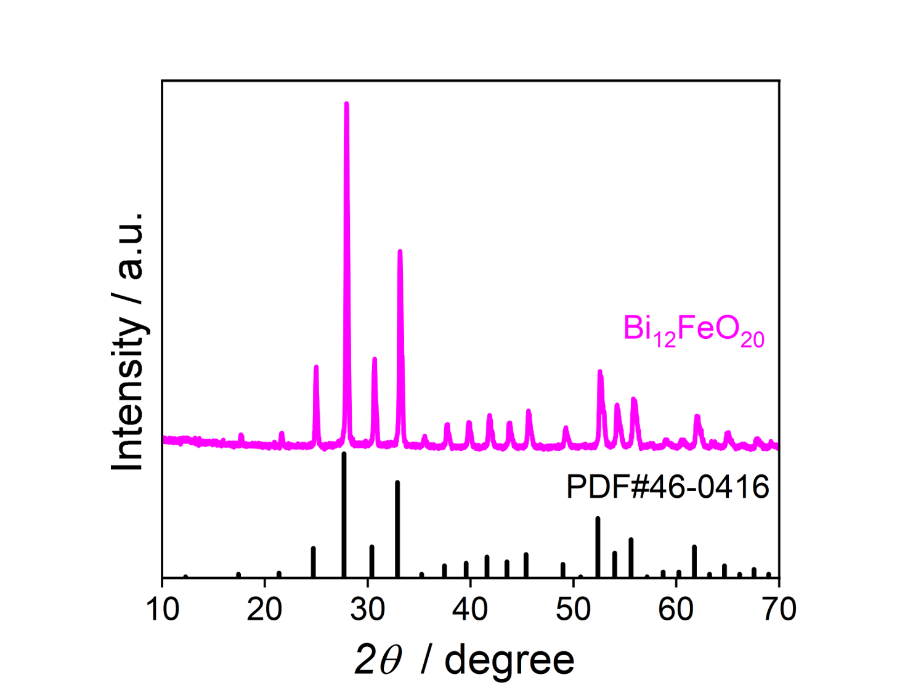


**Figure S1.** XRD patterns of the prepared Bi_12_FeO_20_ nanosheet.

| **Diffractometer** | **Bruker D8** |
| --- | --- |
| Radiation | Cu Kα |
| Range | 10-90° |
| Step | 1°/min |
| Increment | 0.02 |
| Chemical formula | Bi_12.02_Fe_0.98_O_20_ (ICP) |
| Space group | I23 |
| Lattice constant | a=b=c=10.1952 (3) (Å)  α=β=γ=90° |
| Cell Volume (Å^3^) | 1059.7 |
| R_wp_(%) | 9.65 |
| χ^2^(R_wp_/R_ep_) | 2.47 |
| Atomic coordinates | Bi1 (0.182(1) 0.322(2) 0.484(5)),  Bi2 (0, 0, 0), Fe1 (0, 0, 0),  Fe2 (0.182(2) 0.322(7) 0.484(6))  O1 (-0.017(8) 0.279(1) 0.316(4)),  O2 (0.041(7) 0.041(7) 0.041(7)),  O3 (0.193(2) 0.193(2) 0.193(2)) |
| Occupation | Bi1 (0.950), Bi2 (0.414), Fe1 (0.587), Fe2 (0.051), O1 (1.041), O2 (1.069), O3 (1.049) |
| Biso | Bi1 (1.300(2)), Bi2 (6.028(2)), Fe1 (5.810(5)), Fe2 (1.532(4)), O1 (10.435(6)), O2 (6.617(2)), O3 (14.418(6)) |

**Table S1.** XRD refinement parameters for Bi_12_FeO_20_ nanosheet.


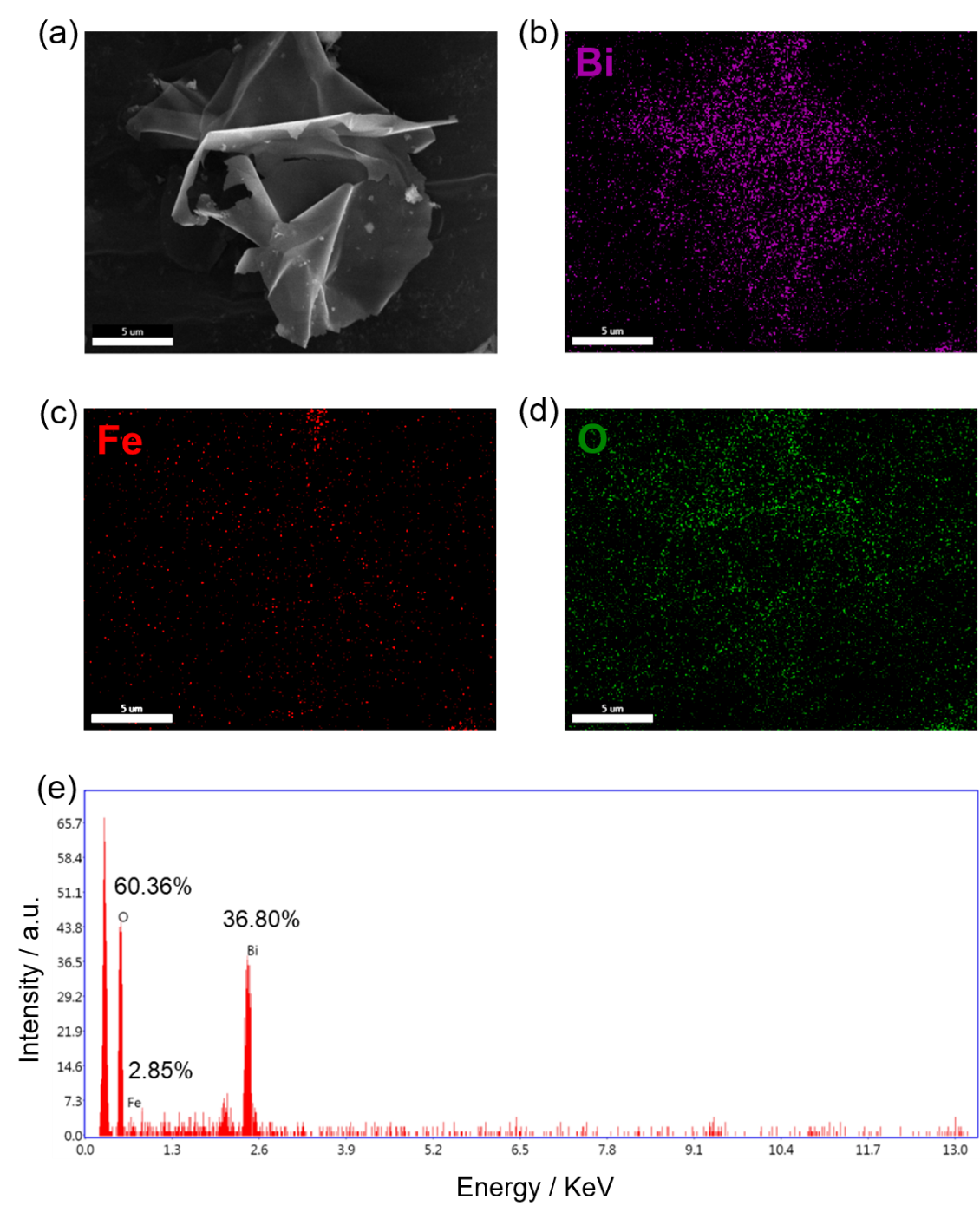


**Figure S2.** SEM-EDX of the prepared Bi_12_FeO_20_ nanosheets. (a) SEM and elemental mapping of (b) Bi, (c) Fe, and (d) O. (e) The concurrent atomic content of Bi, Fe, and O in the Bi_12_FeO_20_ catalyst.


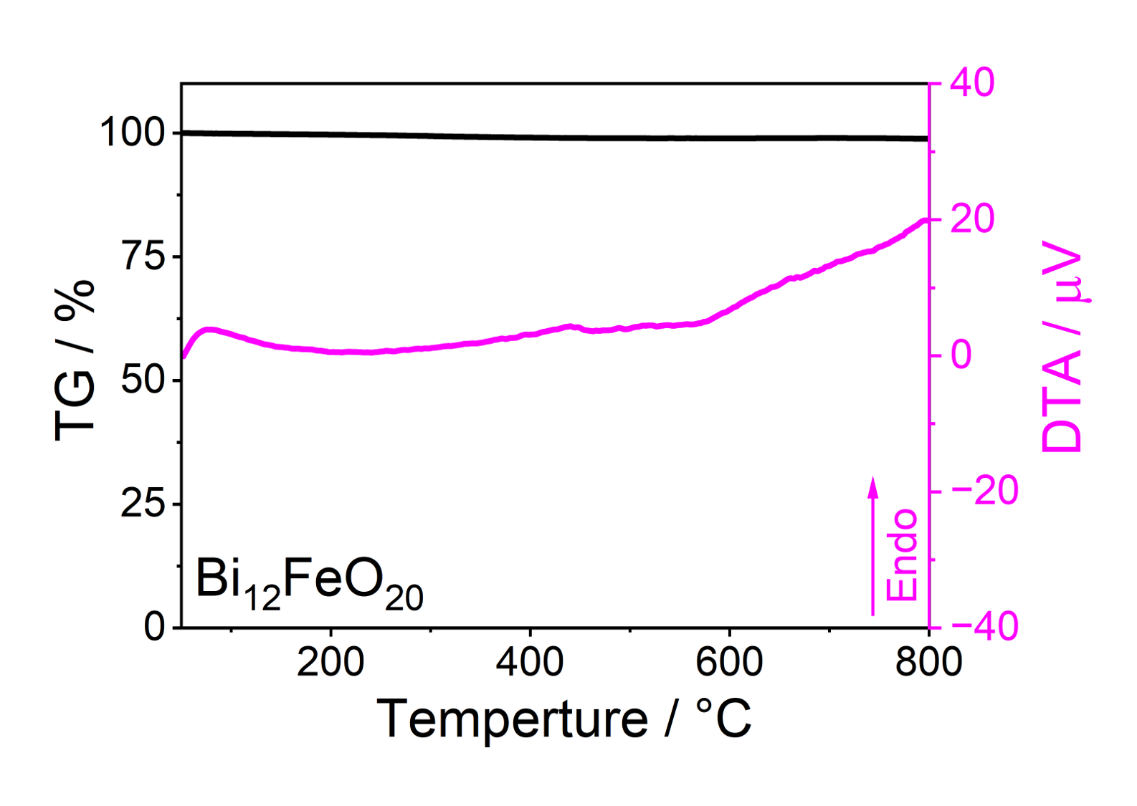


**Figure S3.** Thermogravimetric analysis of the Bi_12_FeO_20_ nanosheet catalyst.


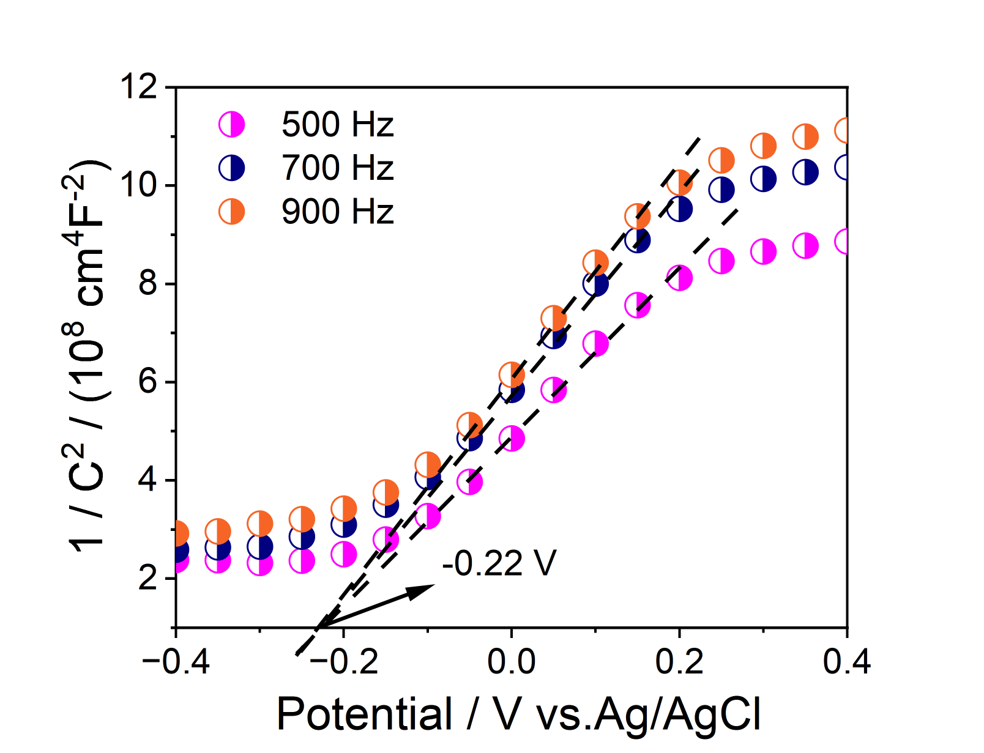


**Figure S4.** The Mott-Schottky curve of the prepared Bi_12_FeO_20_ nanosheets.


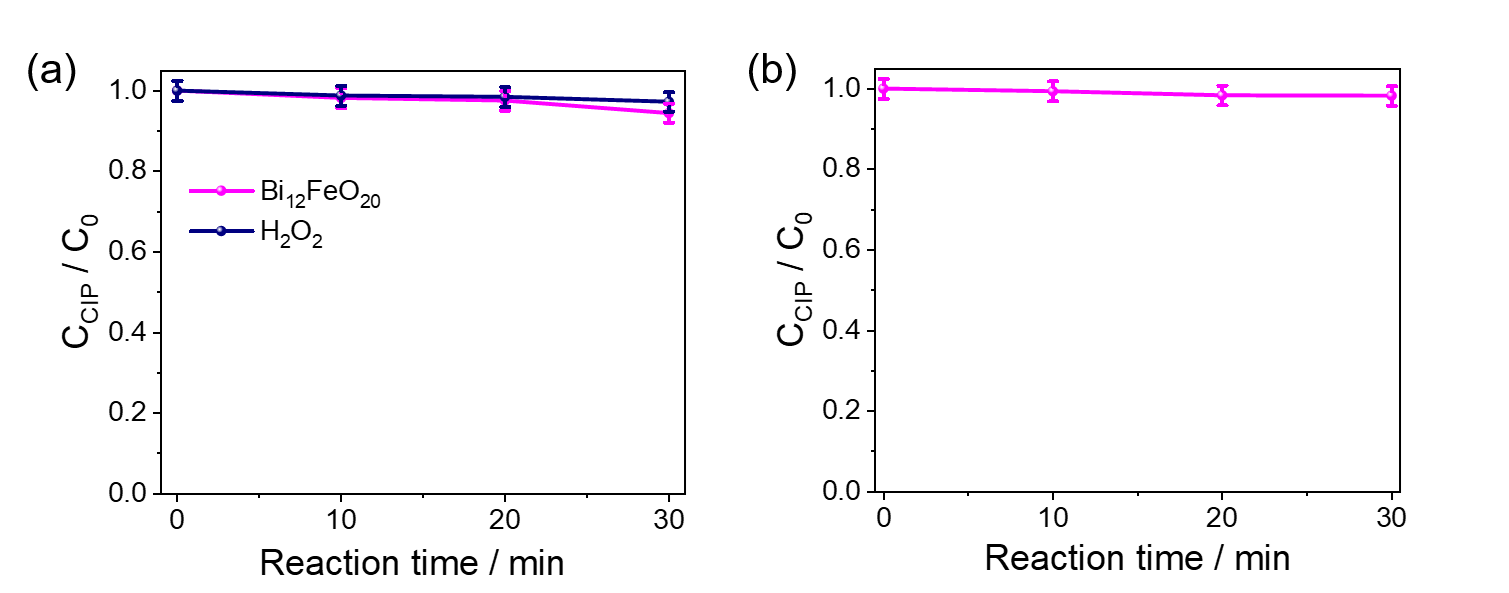


**Figure S5.** (a) The degradation curve of CIP by Bi_12_FeO_20_ and H_2_O_2_ under dark conditions. (b) The photodegradation curve of CIP under irradiation.


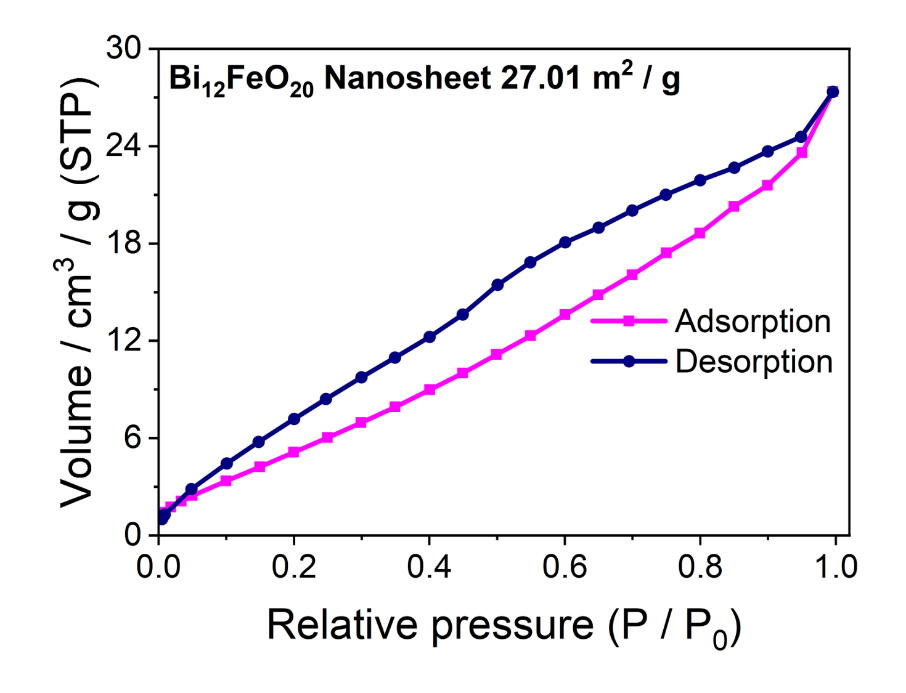


**Figure S6.** Isothermal adsorption-desorption curves of prepared Bi_12_FeO_20_ nanosheets.


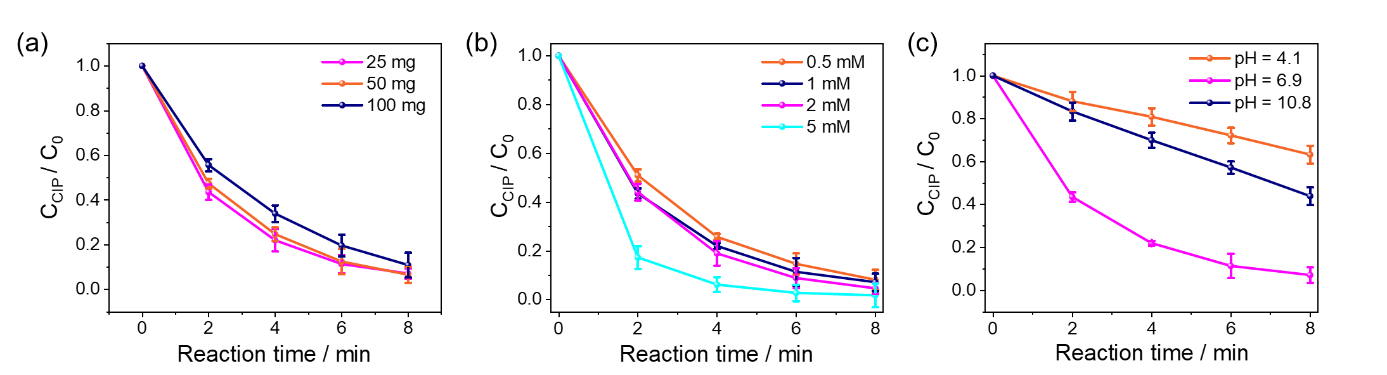
**Figure S7.** (a) Catalyst dosage, (b) H_2_O_2_ concentration, and (c) pH under different conditions affect the degradation curve of CIP. Degradation conditions: [CIP] = 10 mg·L^-1^, Xe lamp λ > 300 nm.

Note that the 5 mM H_2_O_2_ is more effective for CIP degradation, but based on the HPLC spectra, we observed that the generation of the P9 intermediate is similar. Considering factors such as the requirement to minimize residual H_2_O_2_ for subsequent characterizations, we chose 2 mM H_2_O_2_ as the test condition.


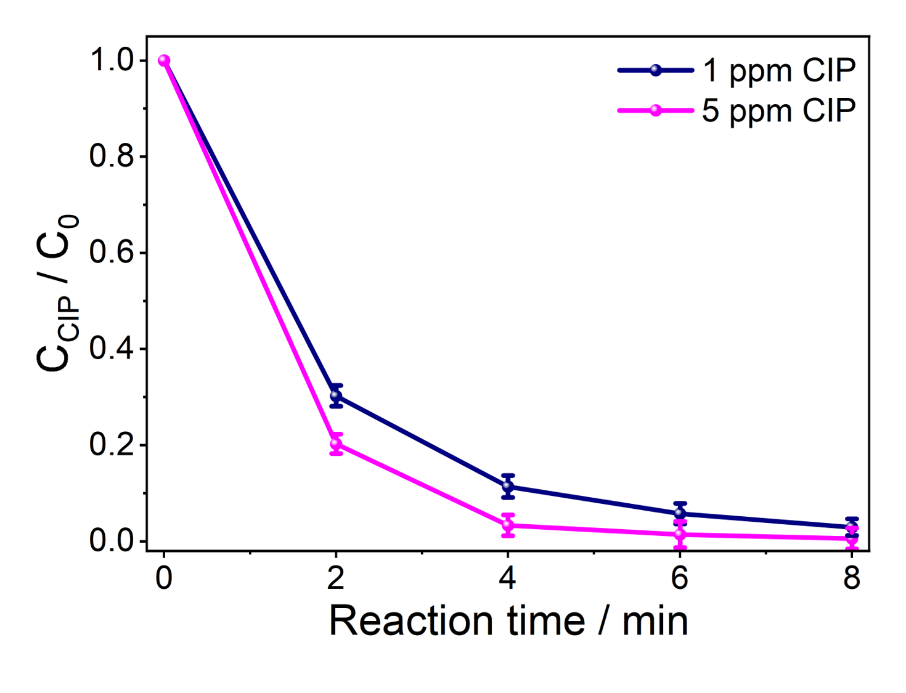


**Figure S8.** Photo-Fenton degradation performance of different CIP concentrations (1 ppm and 5 ppm) in the Bi_12_FeO_20_-H_2_O_2_ system.


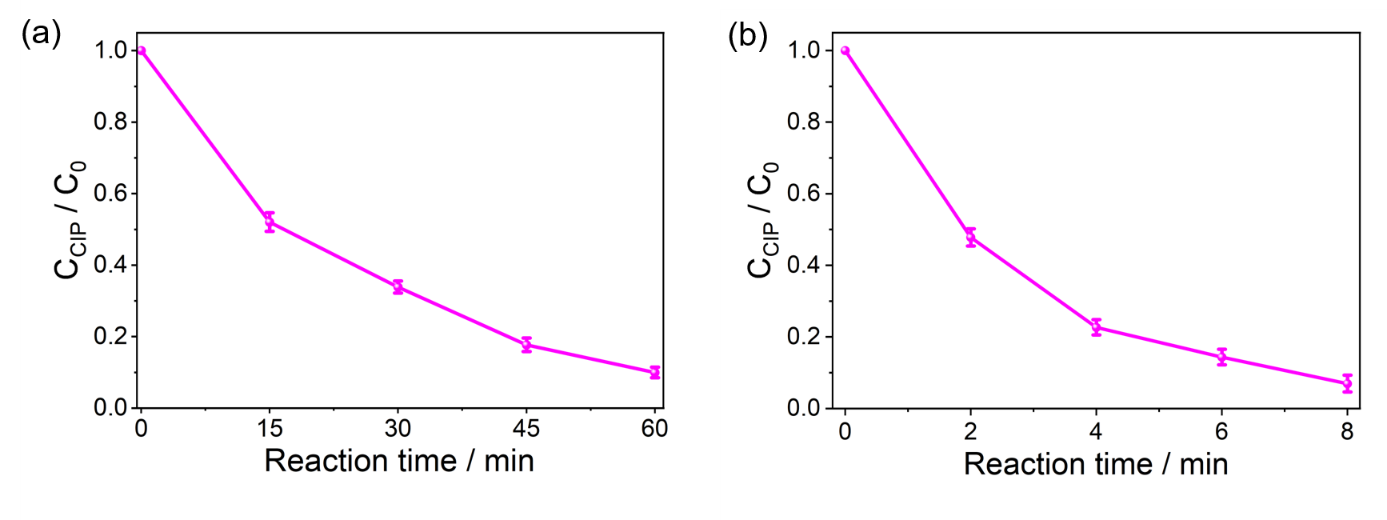


**Figure S9.** (a) The Bi_12_FeO_20_ catalyst degrades CIP in a microbubble system. (b) The Bi_12_FeO_20_ catalyst activates PMS to degrade CIP. Degradation conditions: [CIP]= 10 mg·L^-1^, [Bi_12_FeO_20_]= 0.5 g·L^-1^, [PMS]= 1 mM.


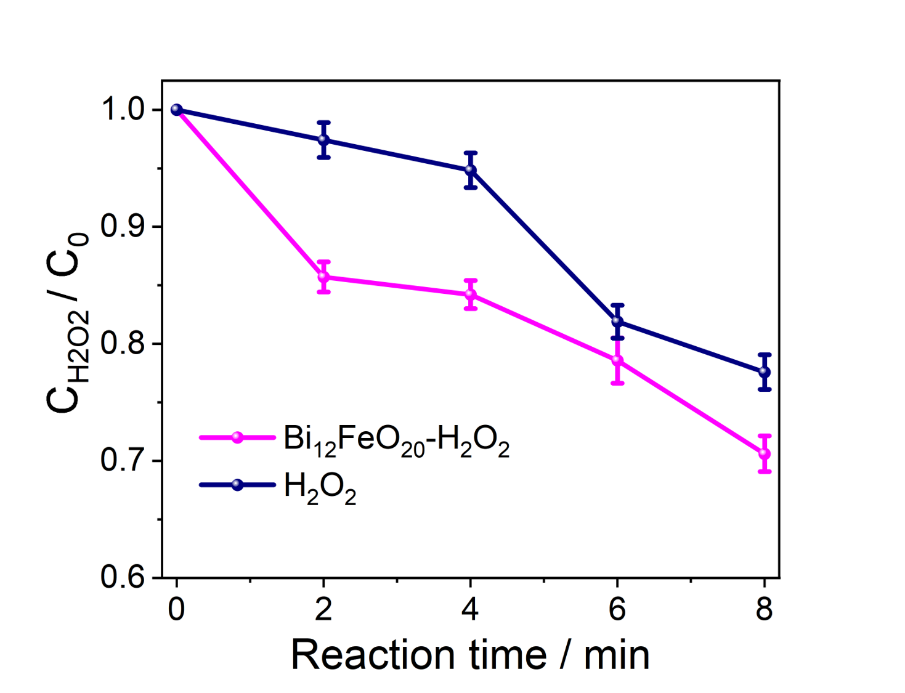


**Figure S10.** The decomposition rate of H_2_O_2_ in the Bi_12_FeO_20_-H_2_O_2_ and H_2_O_2_-alone systems. Reaction conditions: [CIP] = 10 mg·L^-1^, [Bi_12_FeO_20_] = 0.5 g·L^-1^, [H_2_O_2_] = 2 mM, Xe lamp λ > 300 nm.

**Table S2.** The utilization efficiency of H_2_O_2_ in the Bi_12_FeO_20_-H_2_O_2_ and H_2_O_2_-alone systems.

| **Pollutant** | **Systems** | **TOC removal** | **Δ[H_2_O_2_]_s_/mM** | **Δ[H_2_O_2_]_a_/mM** | **η[H_2_O_2_]** |
| --- | --- | --- | --- | --- | --- |
| CIP | H_2_O_2_-alone | 0.2297 | 0.1388 | 0.4483 | 0.3096 |
|  | Bi_12_FeO_20_-H_2_O_2_ | 0.9378 | 0.5667 | 0.5878 | 0.9640 |


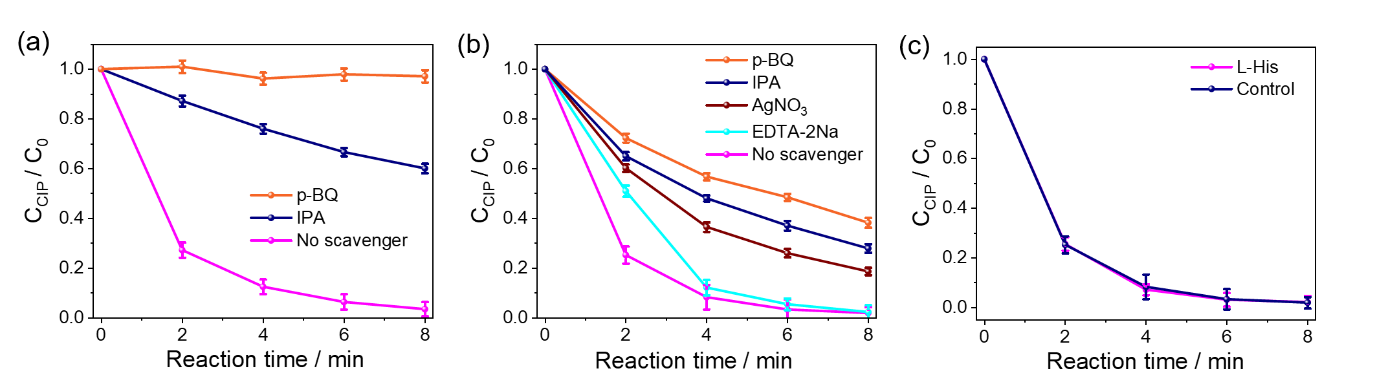


**Figure S11.** (a) Capture experiments in the H_2_O_2_-alone system, and (b) in the Bi_12_FeO_20_-H_2_O_2_ system. (c) Singlet oxygen trapping experiments curve in the Bi_12_FeO_20_-H_2_O_2_ system. Degradation conditions: [CIP] = 10 mg·L^-1^, [Bi_12_FeO_20_] = 0.5 g·L^-1^, [H_2_O_2_] = 2 mM, [IPA] = 5 mM, [p-BQ] = [L-His] = [AgNO_3_] = [EDTA-2Na] = 1 mM, Xe lamp λ > 300 nm.


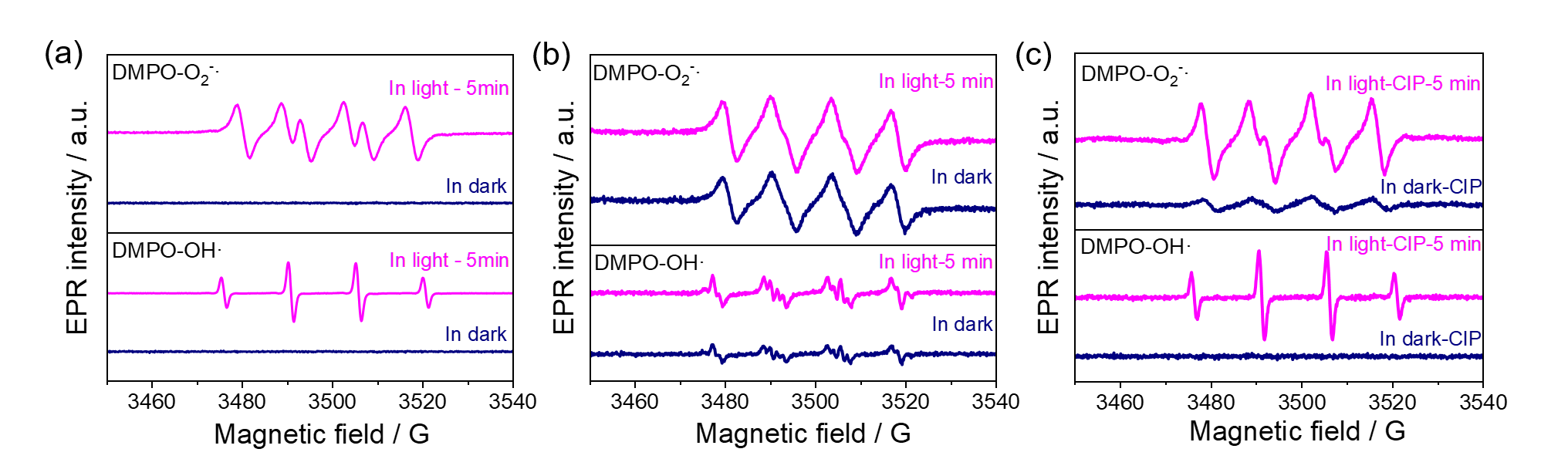


**Figure S12.** (a) EPR spectra of the H_2_O_2_-alone system measured with DMPO as a radical capture reagent. (b) EPR spectra of the Bi_12_FeO_20_-H_2_O_2_ system measured with DMPO as a radical capture reagent. (c) EPR spectra of the Bi_12_FeO_20_-H_2_O_2_ system measured with CIP addition and DMPO as a radical capture reagent. Measurement conditions: [CIP] = 10 mg·L^-1^, [Bi_12_FeO_20_] = 0.5 g·L^-1^, [H_2_O_2_] = 2 mM. The capture ·O_2_^-^ is in methanol, while the capture of ·OH radicals is in water.


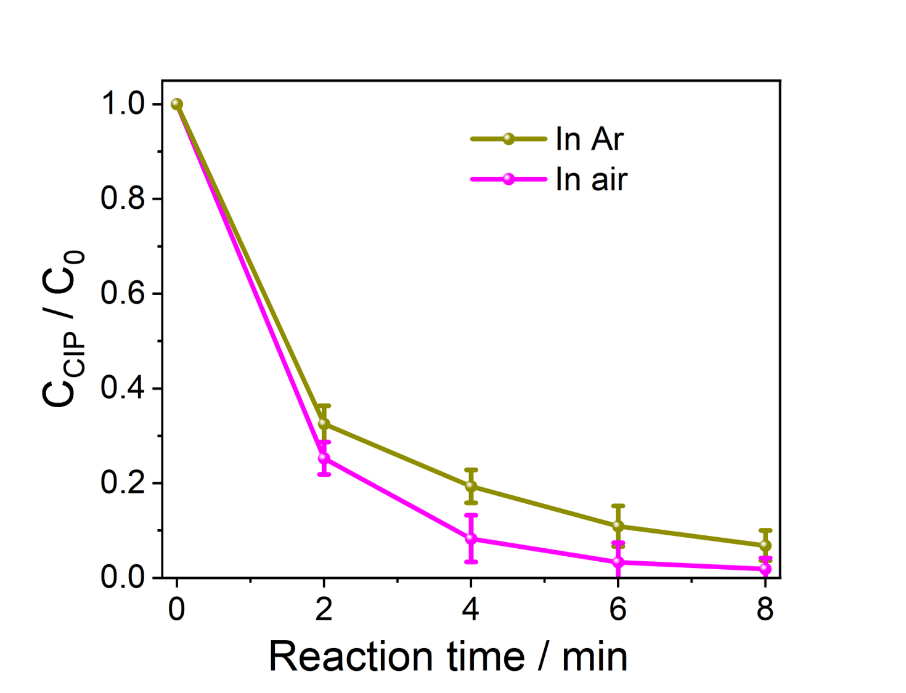


**Figure S13.** The degradation of CIP in the Bi_12_FeO_20_-H_2_O_2_ system in the Ar and air atmosphere. Degradation conditions: [CIP] = 10 mg·L^-1^, [Bi_12_FeO_20_] = 0.5 g·L^-1^, [H_2_O_2_] = 2 mM.


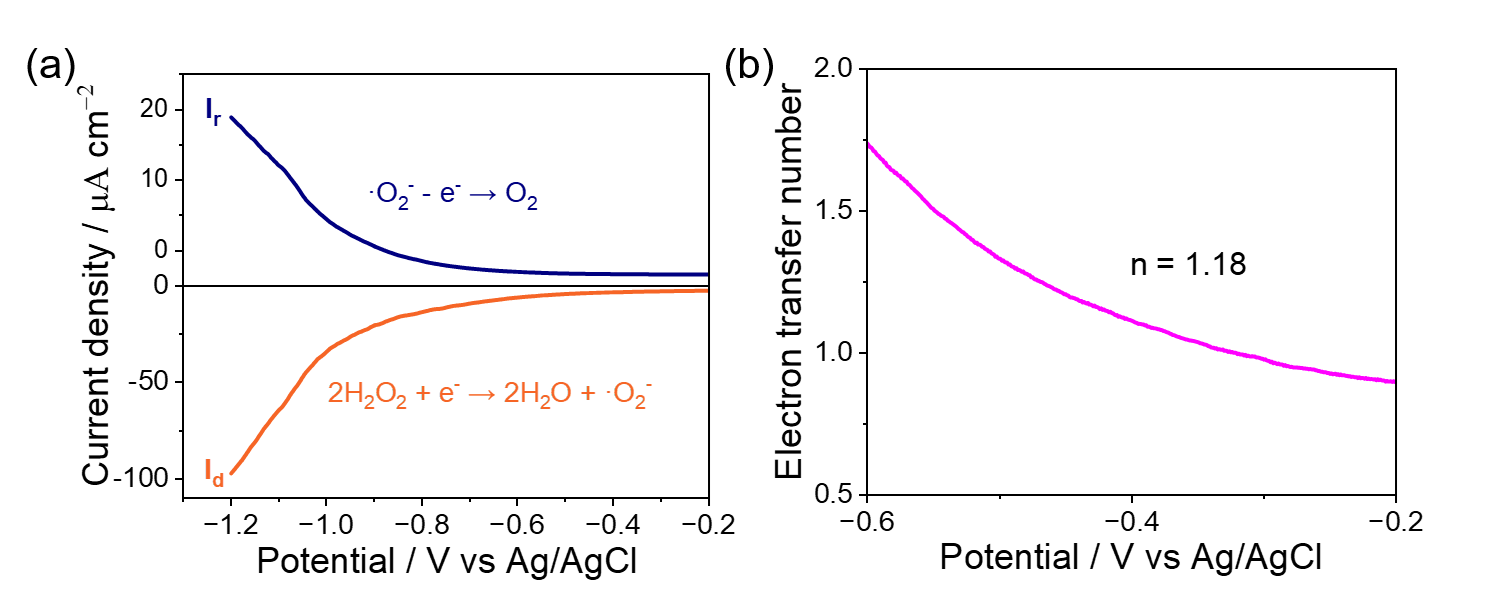


**Figure S14.** (a) RRDE test of the Bi_12_FeO_20_-H_2_O_2_ system and (b) the corresponding electron transfer number.

The RRDE experiments are performed using RRDE-3A (ALS, Japan) and CHI-750E systems (Chenghua Inc., Shanghai, China). The Pt net(1.5 cm × 1.5 cm) serves as the counter electrode, Ag/AgCl acts as the reference electrode, the Bi_12_FeO_20_ / disk electrode (d = 4 mm) as the working electrode, and 0.5 M Na_2_SO_4_ solution functions as the electrolyte. The disk electrode undergoes a cathodic scan at 10 mV·s⁻¹, while the ring electrode is maintained at 0.2 V vs Ag/AgCl and rotates at 1600 rpm in a N_2_ atmosphere. The electron transfer number (n) is determined through the following equation:^[5]^

$n=4\times\frac{I_{d}}{I_{d}+\frac{I_{r}}{N}}$ (Equation S8)

where (I_d_) represents the disk current, (I_r_) denotes the ring current, and (N = 0.38) is the current collection efficiency of the Pt ring.

Due to ·OH's shorter lifetime relative to ·O_2_^-^, ·O_2_^-^ rather than ·OH radicals generated from the H_2_O_2_ activation tend to migrate to the ring electrode to be oxidized. Therefore, RRDE detects ·O_2_^-^ in the Bi_12_FeO_20_-H_2_O_2_ system. The electron transfer number for H_2_O_2_ activation is calculated to be 1.18, indicating a single-electron reduction forming ·O_2_^-^ at the disk.


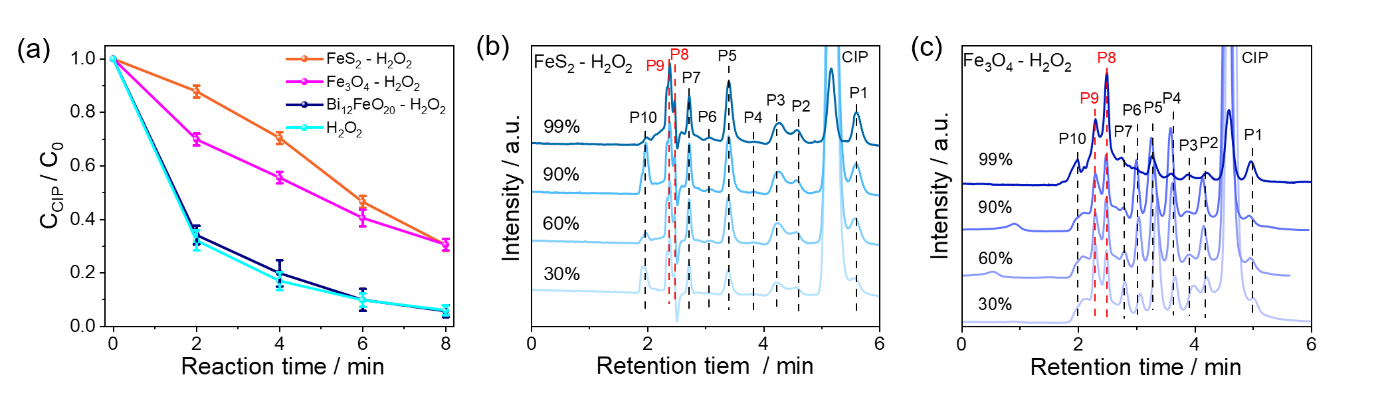


**Figure S15.** (a) The Fenton-like degradation activity for CIP in the FeS_2_-H_2_O_2_, Fe_3_O_4_-H_2_O_2_, Bi_12_FeO_20_-H_2_O_2_, and H_2_O_2_-alone systems. (b) The HPLC spectra of CIP degradation by 30% (2 min), 60% (4 min), 90% (8 min), and 99% (30 min) in the FeS_2_-H_2_O_2_ system. (c) The HPLC spectra of CIP derogation by 30% (2 min), 60% (6 min), 90% (8 min), and 99% (20 min) in the Fe_3_O_4_-H_2_O_2_ system. Degradation conditions: [CIP] = 10 mg·L^-1^, [Bi_12_FeO_20_] = [FeS_2_] = [Fe_3_O_4_] = 0.5 g·L^-1^, [H_2_O_2_] = 2 mM.


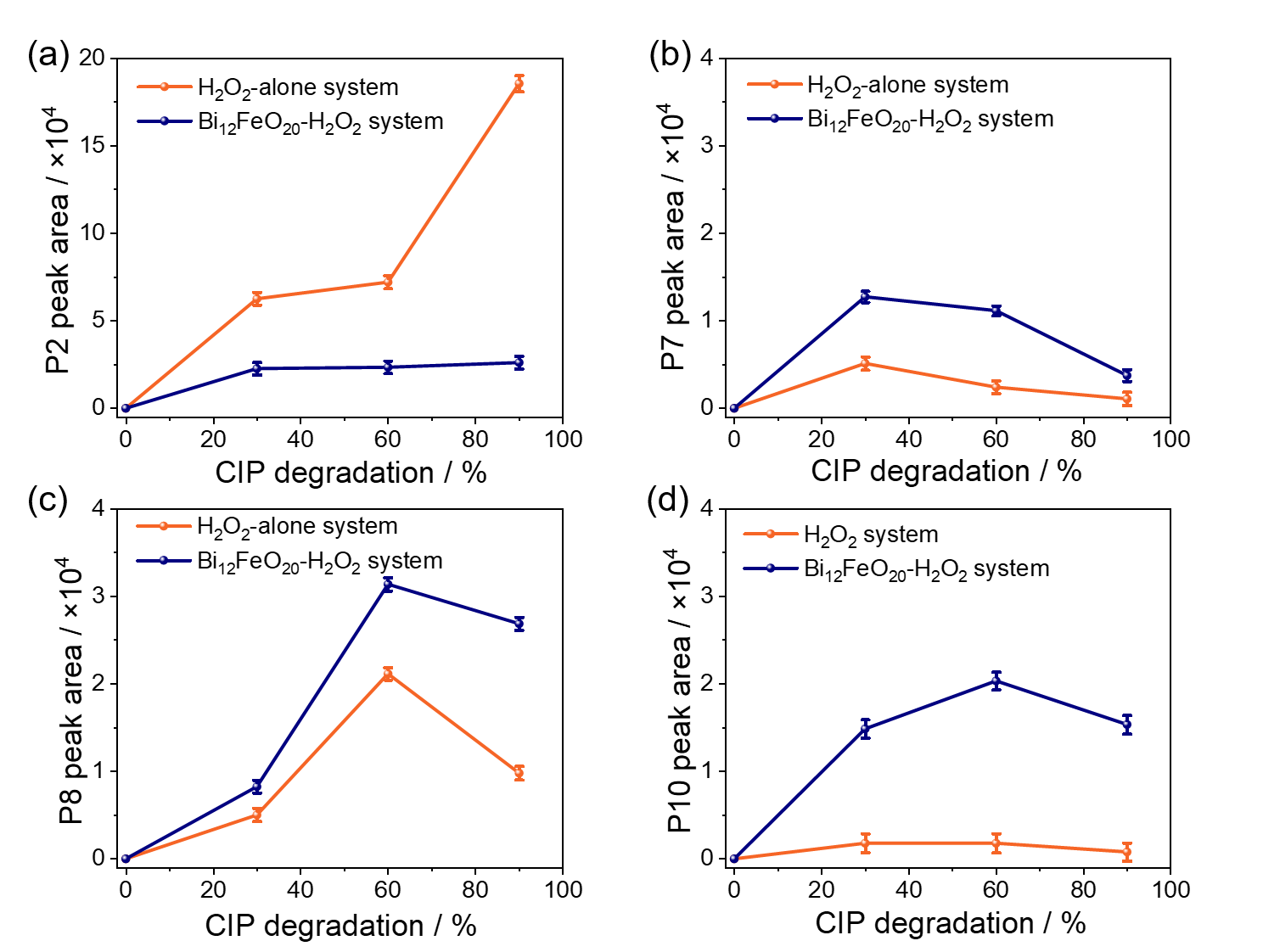


**Figure S16.** The peak area change of (a) the P2 intermediate, (b) the P7 intermediate, (c) the P8 intermediate, and (d) the P10 intermediate versus the CIP degradation percentage in the Bi_12_FeO_20_-H_2_O_2_ and H_2_O_2_-alone systems.


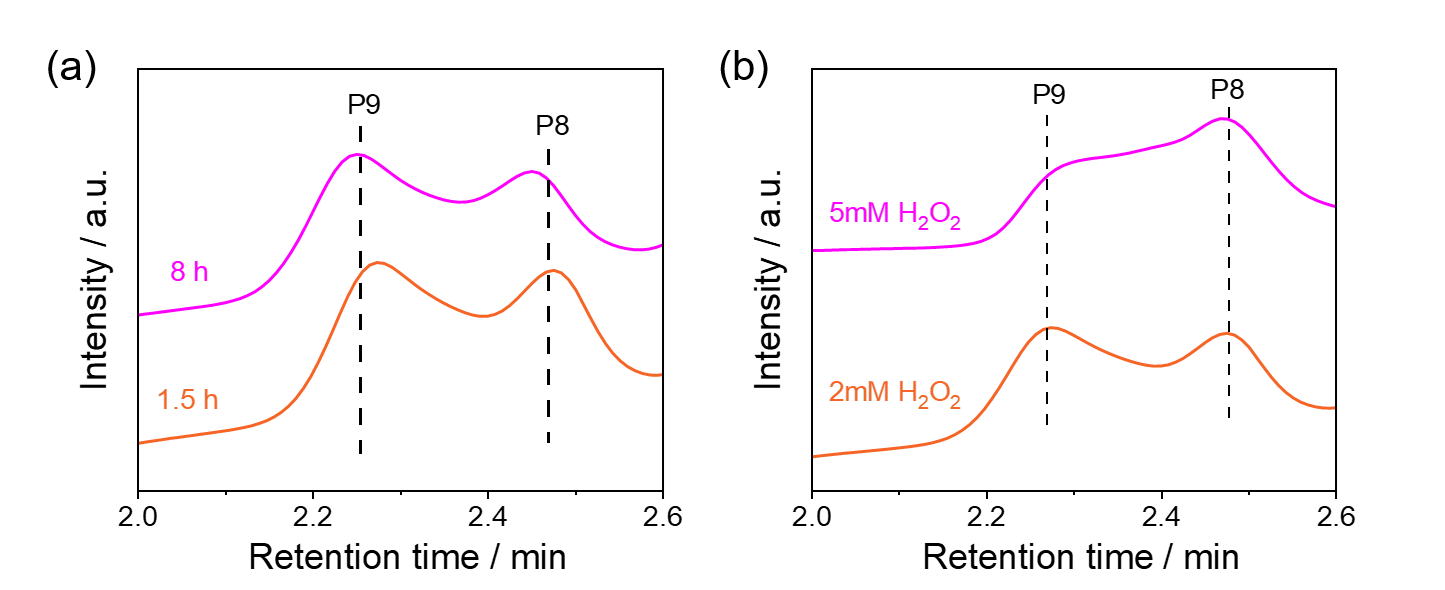


**Figure S17.** The HPLC spectra of 8 h CIP degradation (a) and with 5 mM H_2_O_2_ added in the H_2_O_2_-alone system. Degradation conditions: [CIP] = 10 mg·L^-1^.


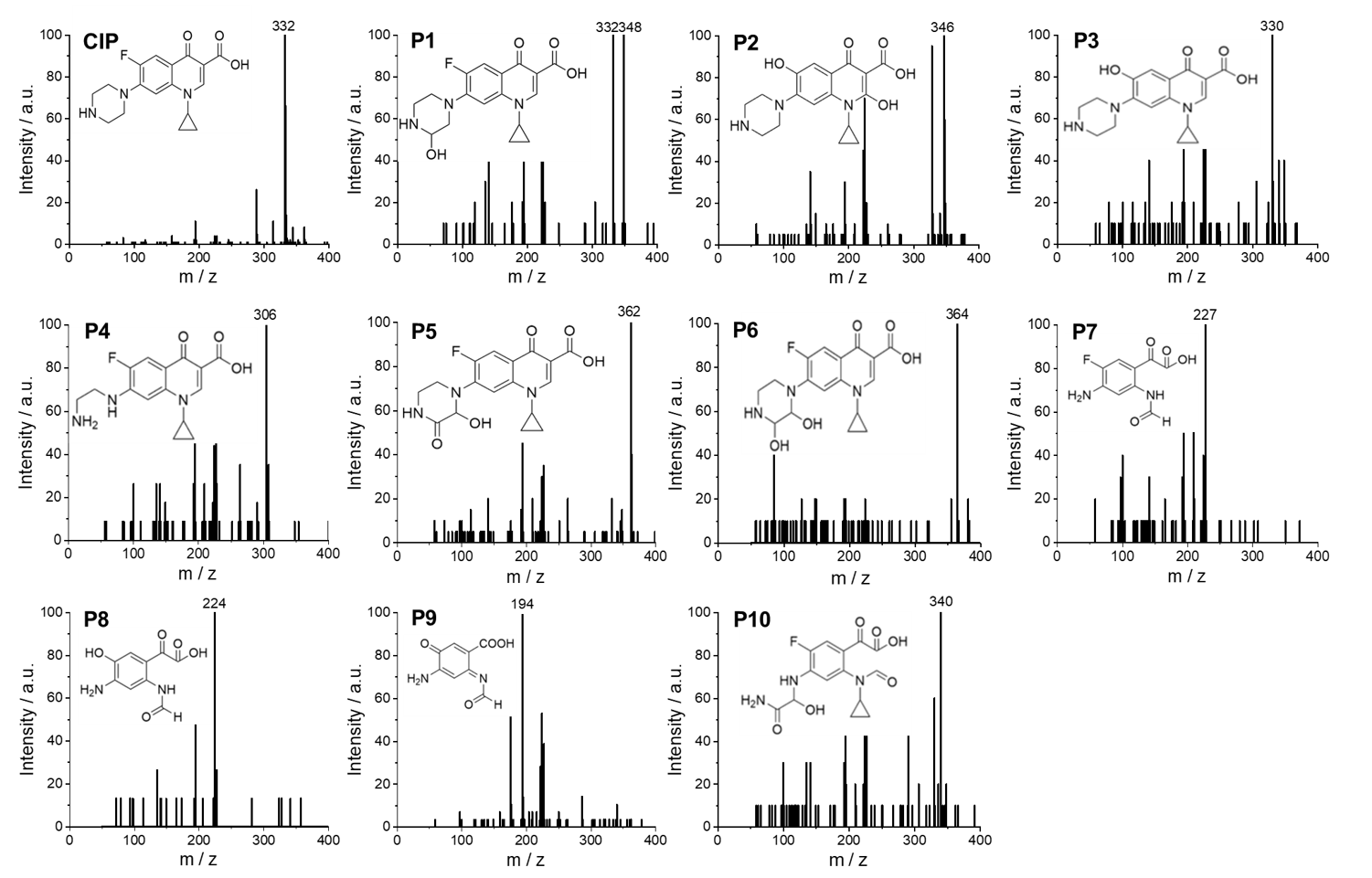


**Figure S18.** The LC-MS spectra of the CIP and the intermediates (P1-P10) produced by CIP degradation.

| **Compound** | | **t_R_/min** | **ESI(+) MS/MS (m/z)** | **Molecular formula** | **Chemical structure** |
| --- | --- | --- | --- | --- | --- |
| CIP | 5.19 | | 332 | C_17_H_18_FN_3_O_3_ | **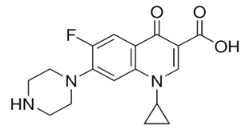** |
| P1 | 5.72 | | 348 | C_17_H_18_FN_3_O_4_ | **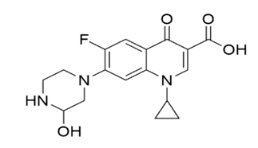** |
| P2 | 4.75 | | 346 | C_17_H_19_N_3_O_5_ | **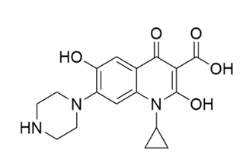** |
| P3 | 4.26 | | 330 | C_17_H_19_N_3_O_4_ | **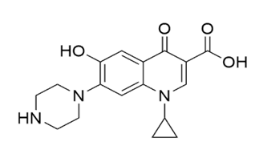** |
| P4 | 3.94 | | 306 | C_15_H_16_FN_3_O_3_ | **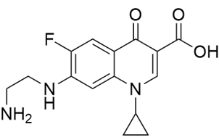** |
| P5 | 3.50 | | 362 | C_17_H_16_FN_3_O_5_ | **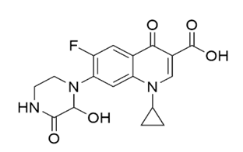** |
| P6 | 3.13 | | 364 | C_17_H_18_FN_3_O_5_ | **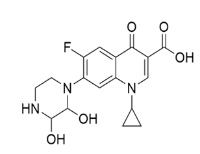** |
| P7 | 2.81 | | 227 | C_9_H_7_FN_2_O_4_ | **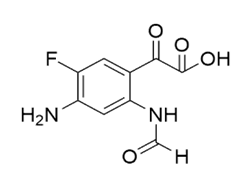** |
| P8 | 2.45 | | 224 | C_9_H_8_N_2_O_5_ | **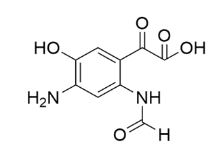** |
| P9 | 2.33 | | 194 | C_8_H_6_N_2_O_4_ | **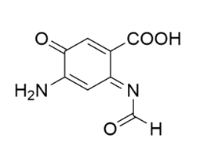** |
| P10 | 2.00 | | 340 | C_14_H_14_FN_3_O_6_ | **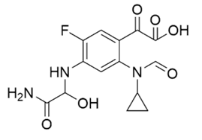** |

**Table S3.** The systematic analysis and identification of CIP and its degradation intermediates (P1-P10) are conducted about the retention time (t_R_), m/z, molecular formula, and proposed chemical structures.


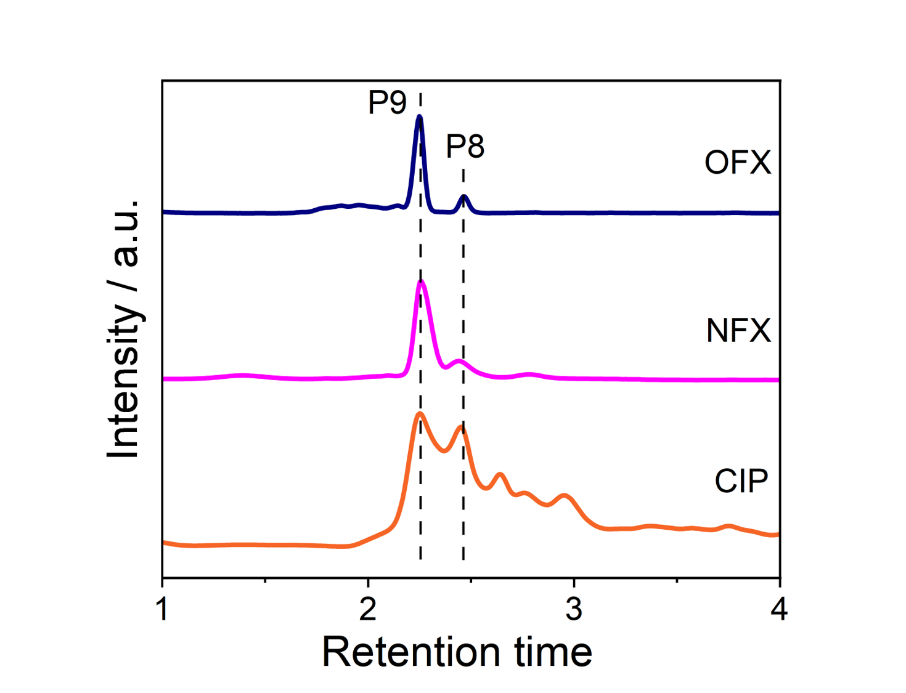


**Figure S19.** The HPLC spectra of 8 h CIP degradation, NFX degradation, and OFX degradation in the H_2_O_2_-alone system. Degradation conditions: [CIP] = [NFX] = [OFX] = 10 mg·L^-1^.

**Table S4.** The toxicity assessment levels.

| **Toxicity range (mg/L)** | **Toxicity level** |
| --- | --- |
| LC_50_/EC_50_/ChV ≤ 1 | Highly toxic |
| 1 < LC_50_/EC_50_/ChV ≤ 10 | Toxic |
| 10 < LC_50_/EC_50_/ChV ≤ 100 | Harmful |
| LC_50_/EC_50_/ChV > 10 | Not harmful |


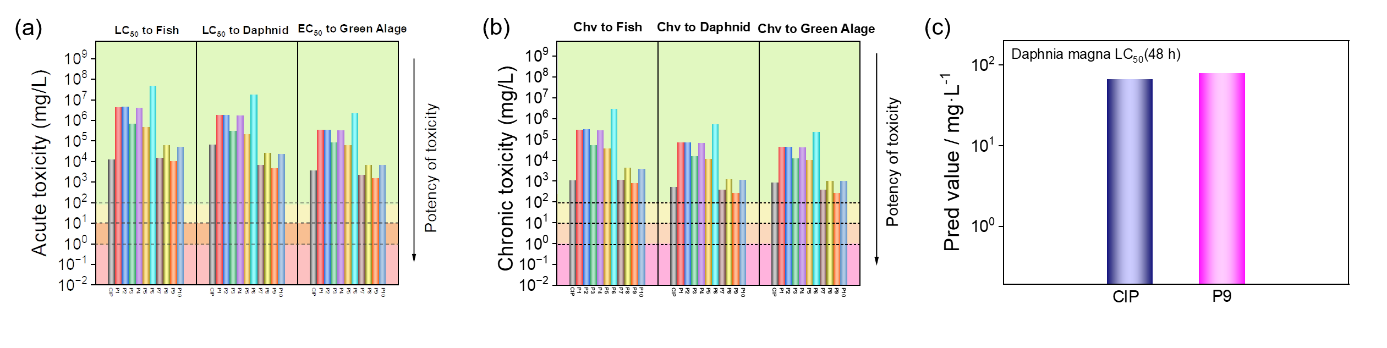


**Figure S20.** (a) The acute and (b) chronic toxicity of CIP and intermediates (P1-P10) to fish, daphnid, and green algae. The ECOSAR software performs the toxicity prediction. (c) The predicted toxicity of CIP and quinone-imine (P9) intermediates to daphnid magna (48 h). The T.E.S.T. software performs the toxicity prediction.


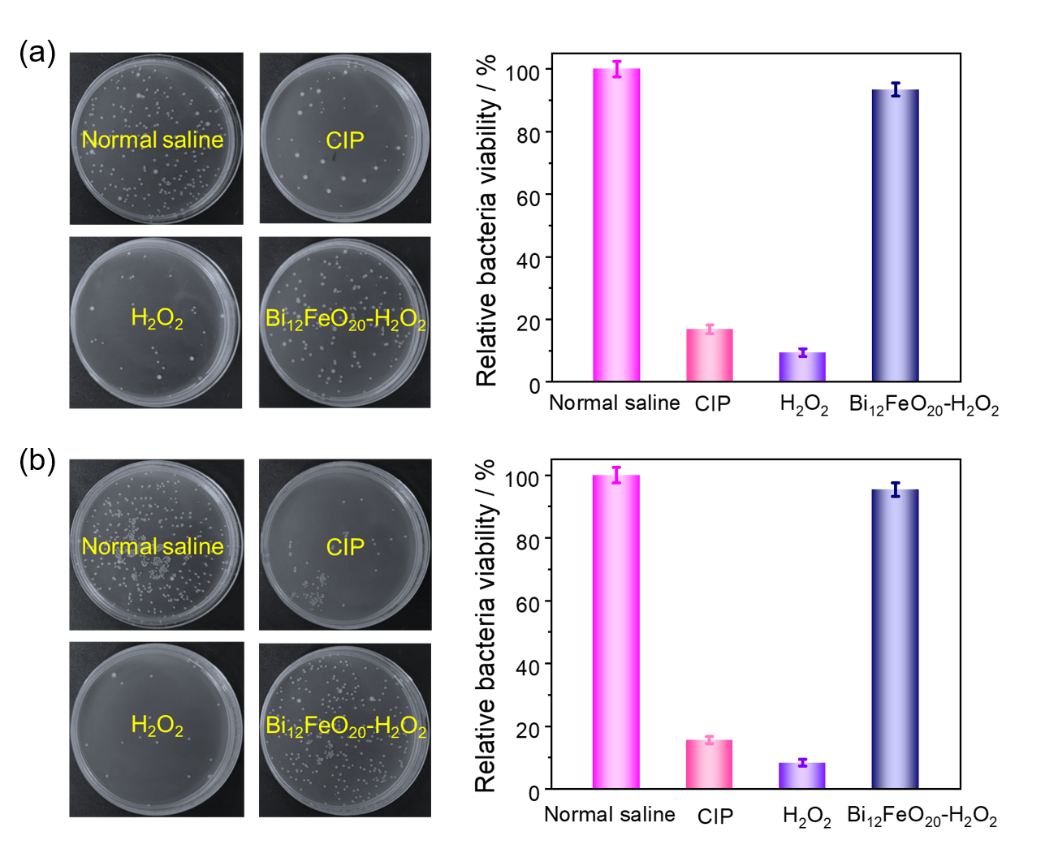


**Figure S21.** (a) The colony images and the relative bacterial viability of the toxicity test using Escherichia coli O157 with the untreated and treated 1 ppm CIP solution in the Bi_12_FeO_20_-H_2_O_2_ and H_2_O_2_-alone systems. (b) And the untreated and treated 0.1 ppm CIP solutions in the Bi_12_FeO_20_-H_2_O_2_ and H_2_O_2_-alone systems. The normal saline is used as the control.

**Table S5.** The growth patterns of soybean sprouts under various conditions.

| **Time/day** | **Average length/cm** | **Tap water** | **CIP** | **H_2_O_2_-alone system** | **Bi_12_FeO_20_-H_2_O_2_ system** |
| --- | --- | --- | --- | --- | --- |
| 1 | stem | 0.95 ± 0.1 | 0.45 ± 0.05 | 0.30 ± 0.01 | 0.90 ± 0.1 |
|  | root | 0 ± 0 | 0 ± 0 | 0 ± 0 | 0 ± 0 |
| 2 | stem | 2.00 ± 0.1 | 0.80 ± 0.05 | 0.75 ± 0.02 | 1.90 ± 0.2 |
|  | root | 0 ± 0 | 0 ± 0 | 0 ± 0 | 0 ± 0 |
| 3 | stem | 2.65 ± 0.2 | 1.15 ± 0.05 | 1.05 ± 0.05 | 2.60 ± 0.3 |
|  | root | 0 ± 0 | 0 ± 0 | 0 ± 0 | 0 ± 0 |
| 4 | stem | 5.10 ± 0.3 | 1.35 ± 0.06 | 1.30 ± 0.05 | 4.25 ± 0.2 |
|  | root | 1.45 ± 0.05 | 0 ± 0 | 0 ± 0 | 0.50 ± 0.03 |
| 5 | stem | 10.00 ± 0.5 | 1.65 ± 0.04 | 1.50 ± 0.02 | 7.50 ± 0.8 |
|  | root | 4.20 ± 0.08 | 0 ± 0 | 0 ± 0 | 1.85 ± 0.02 |
| 6 | stem | 14.30 ± 0.6 | 2.15 ± 0.02 | 1.75 ± 0.02 | 12.45 ± 0.2 |
|  | root | 6.90 ± 0.1 | 0 ± 0 | 0 ± 0 | 6.05 ± 0.1 |


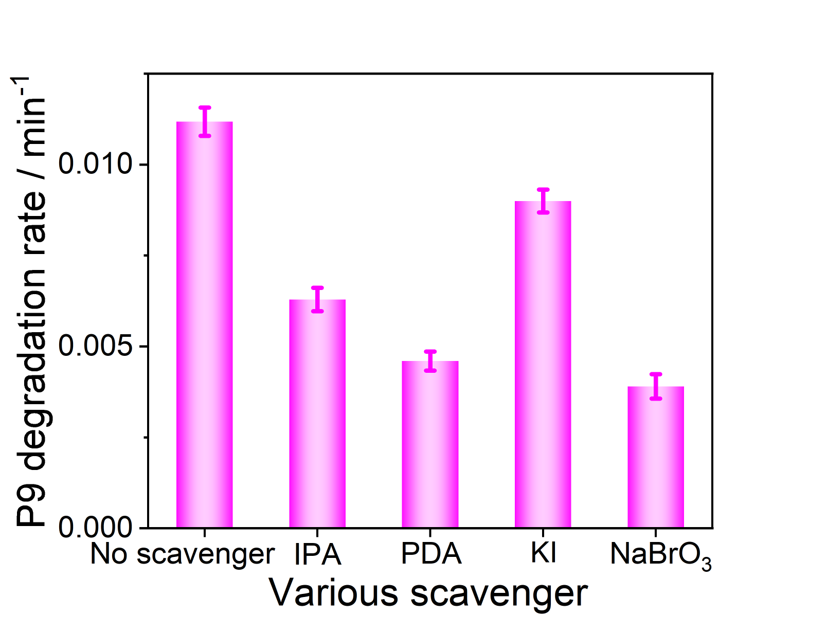


**Figure S22.** The degradation rate constant of the quinone-imine intermediates (P9) in the Bi_12_FeO_20_-H_2_O_2_ system with different capture reagents. Degradation conditions: [Bi_12_FeO_20_] = 0.5 g·L^-1^, [H_2_O_2_] = 2 mM, [IPA] = 5 mM, [KI] = [NaBrO_3_] = 1 mM, [PDA] = 10 ppm.


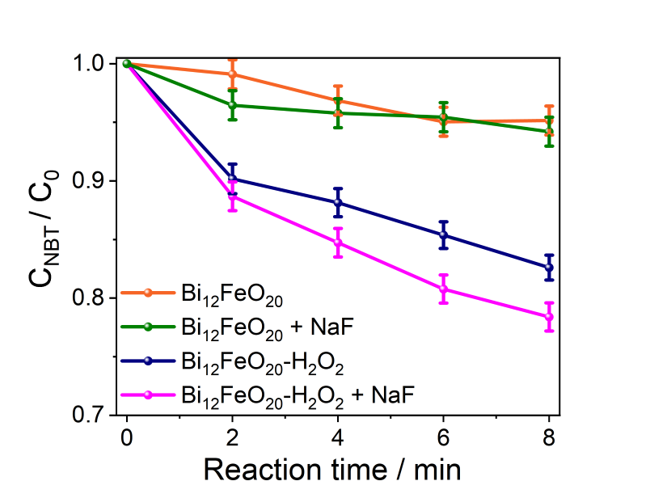


**Figure S23.** Comparison of the NBT consumption rate in the Bi_12_FeO_20_-alone and Bi_12_FeO_20_-H_2_O_2_ with and without NaF addition. Reaction conditions: [CIP] = 10 mg·L^-1^,[Bi_12_FeO_20_] = 0.5 g·L^-1^, [H_2_O_2_] = 2 mM, [NaF] = 50 mM, [NBT] = 0.01 mM.


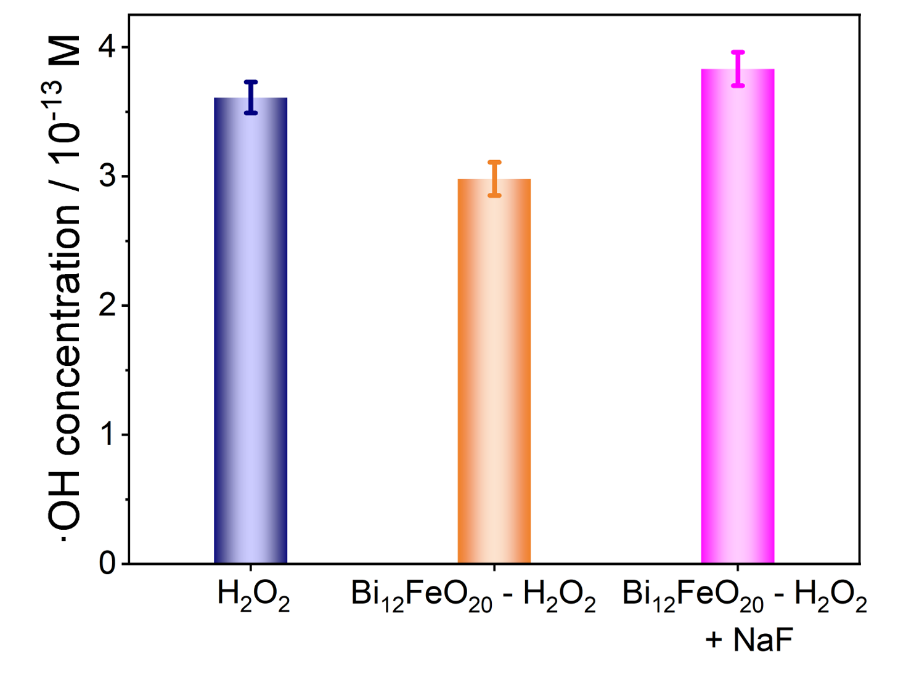


**Figure S24.** Comparison of the ·OH concentration in the H_2_O_2_-alone and Bi_12_FeO_20_-H_2_O_2_ with and without NaF addition.


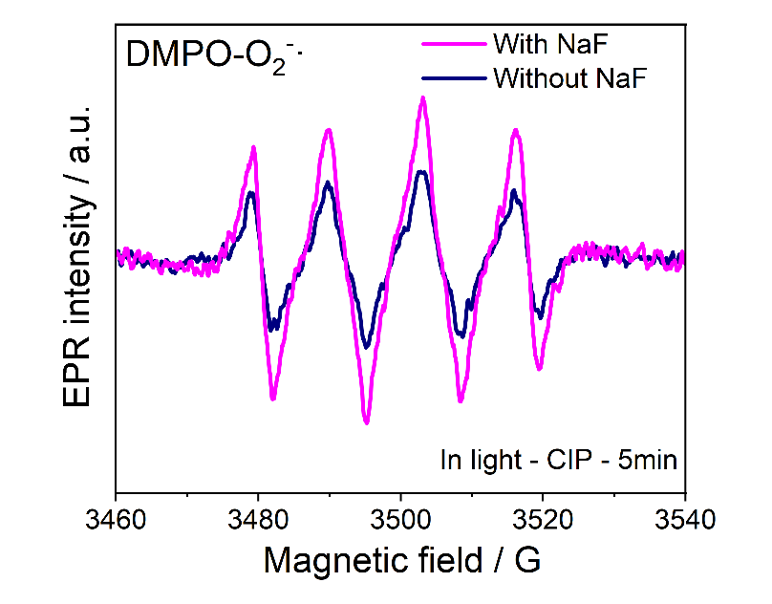


**Figure S25.** EPR spectra of the DMPO-O_2_^-^· signals measured in the Bi_12_FeO_20_-H_2_O_2_ system with and without NaF addition in light 5min.


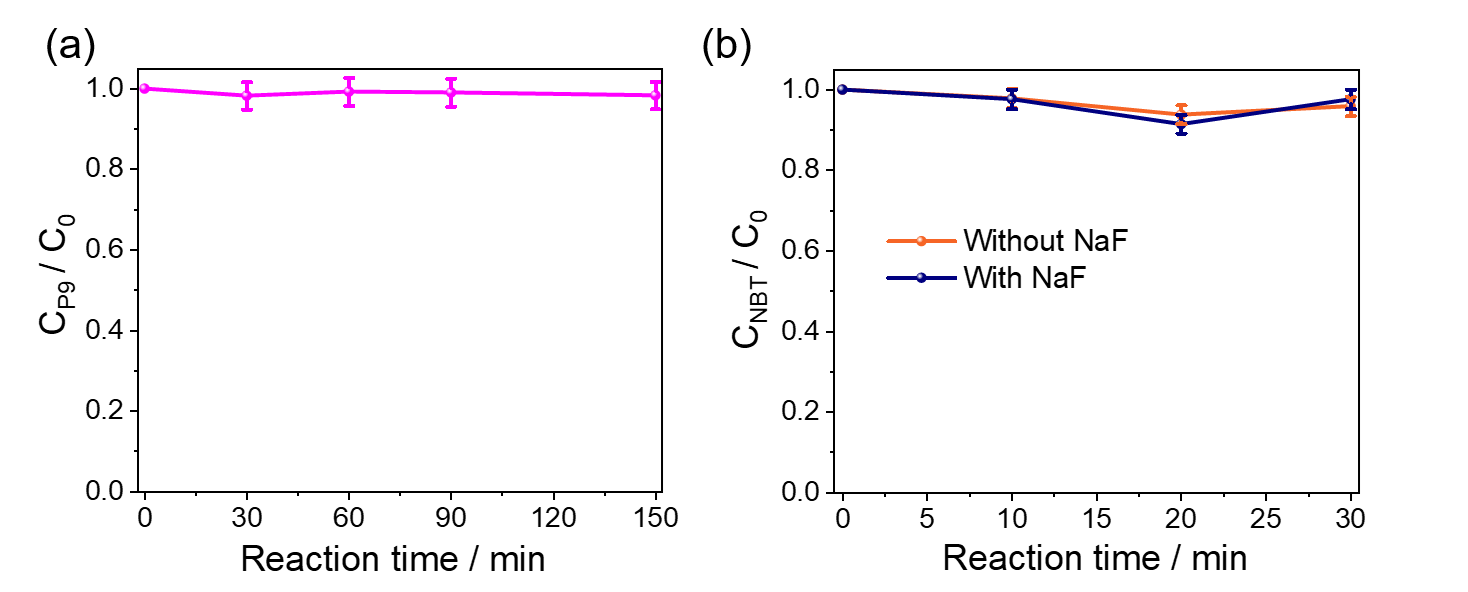


**Figure S26.** (a) The degradation rate of quinone-imine intermediate (P9) by pyrite (FeS_2_) is the Fenton catalyst. (b) The NBT consumption in the FeS_2_-H_2_O_2_ system with and without NaF addition. The decrease in NBT concentration is equal to the generation rate of ·O_2_^-^. Reaction conditions: [CIP] = 10 mg·L^-1^, [H_2_O_2_] = 2 mM, [FeS_2_] = 0.5 g·L^-1^, [NaF] = 50 mM.


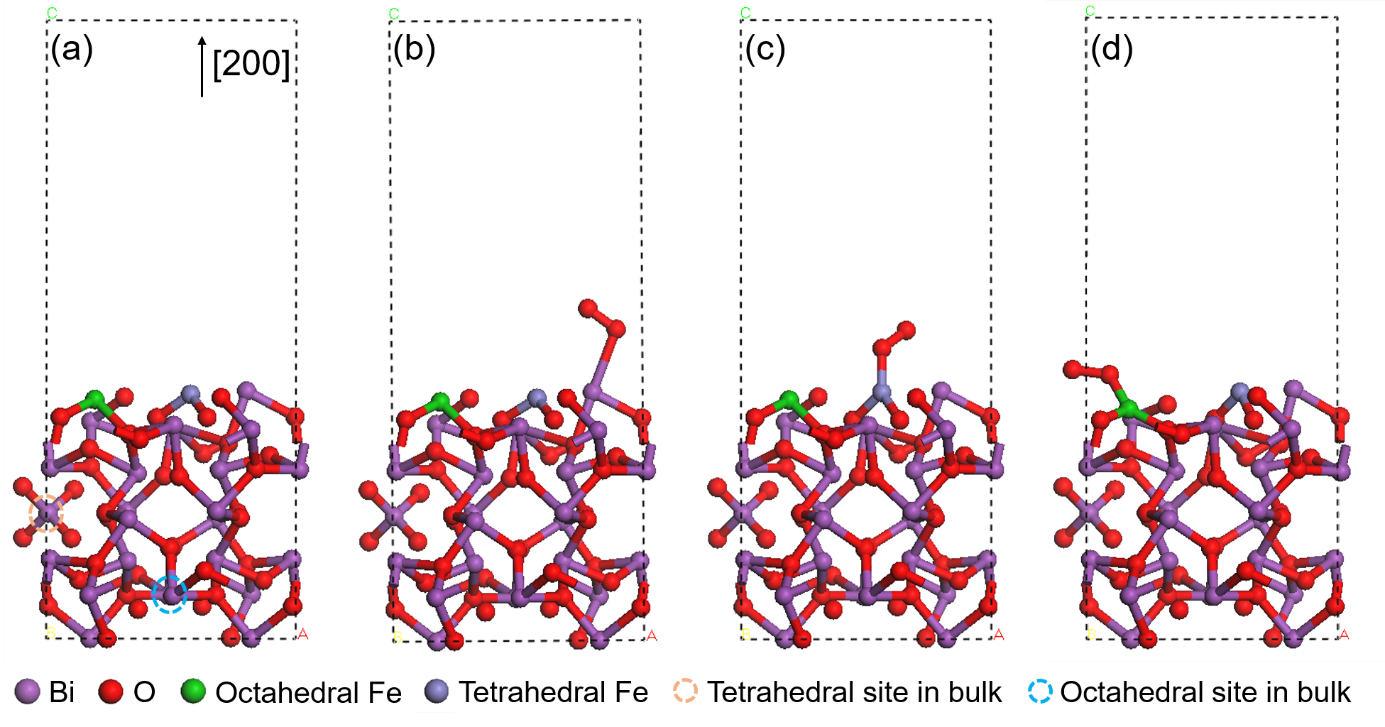


**Figure S27.** (a) The optimized structure of the (200) surface of Bi_12_FeO_20_. Structural models for ·O_2_^-^ adsorption on the Bi site (Bi*O_2_^-^) (b), the tetrahedral Fe site (Fe_tet_*O_2_^-^) (c), and the octahedral Fe site (Fe_oct_*O_2_^-^). In the sillenite structure, metal cations are known to occupy either tetrahedral (orange) or octahedral (blue) sites in the bulk, as indicated by the hollow circles. Notably, the Fe atoms at both tetrahedral and octahedral sites are undercoordinated relative to their bulk counterparts because they are at the surface.


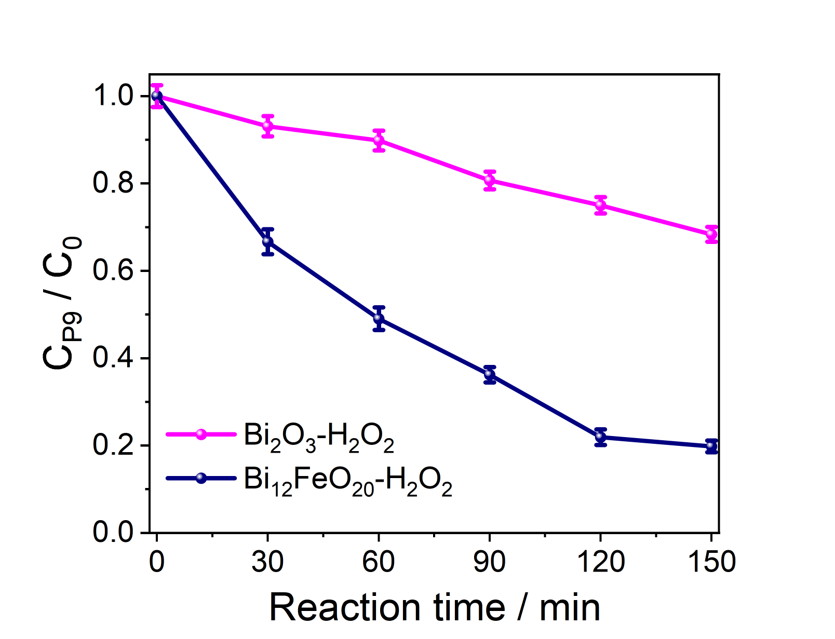


**Figure S28.** Degradation curves of quinone-imine intermediates (P9) in the Bi_12_FeO_20_-H_2_O_2_ and Bi_2_O_3_-H_2_O_2_ systems. Reaction conditions: [CIP] = 10 mg·L^-1^, [H_2_O_2_] = 2 mM, [Bi_12_FeO_20_] = [Bi_2_O_3_] = 0.5 g·L^-1^.


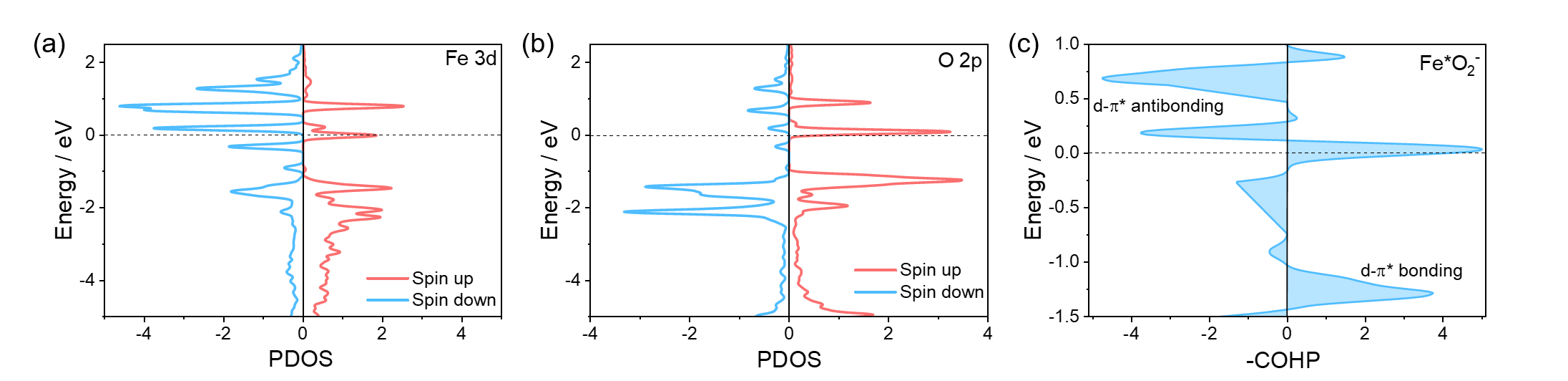


**Figure S29.** The partial density of states (PDOS) calculations for Bi_12_FeO_20_ are presented for (a) Fe, (b) O, and (c) Fe*O_2_^-^.


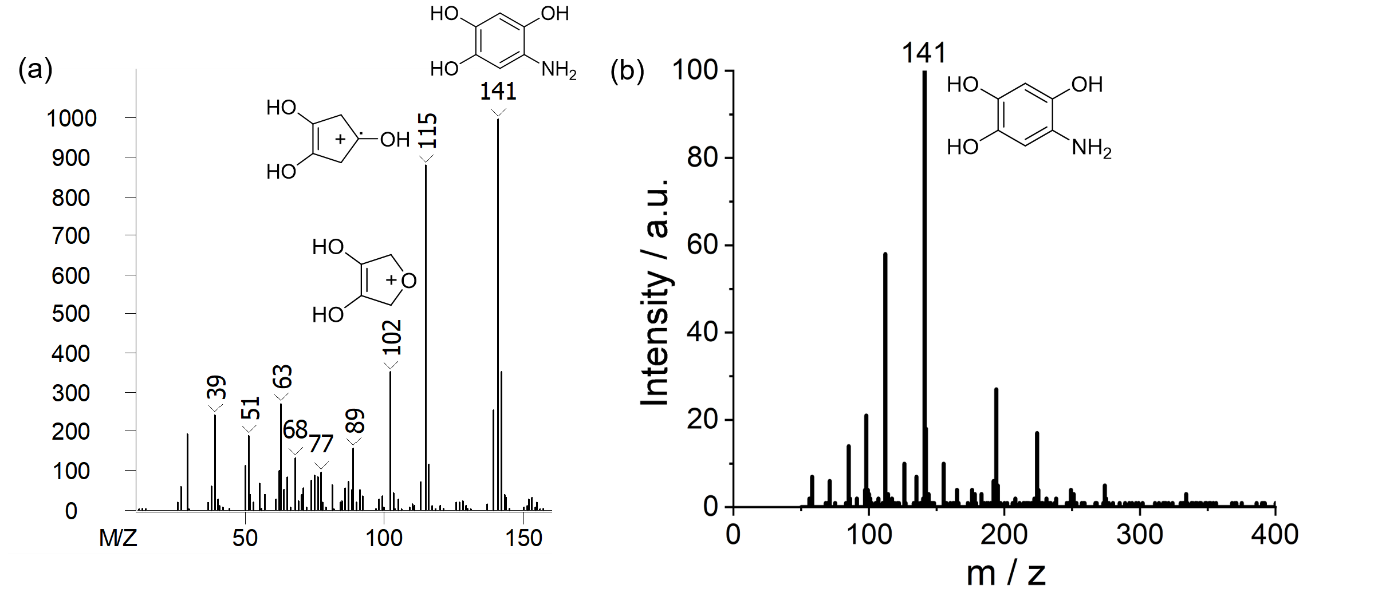


**Figure S30.** (a) The GC-MS spectrum of the m/z =141 intermediate generated from the further degradation of the quinone-imine intermediate with surface-bound ·O_2_^-^. The possible structure of characteristic m/z = 115 and 102 fragments derived from the m/z = 141 compound is also shown inset. (b) The LC-MS spectrum of the intermediates m/z = 141 produced by CIP degradation.


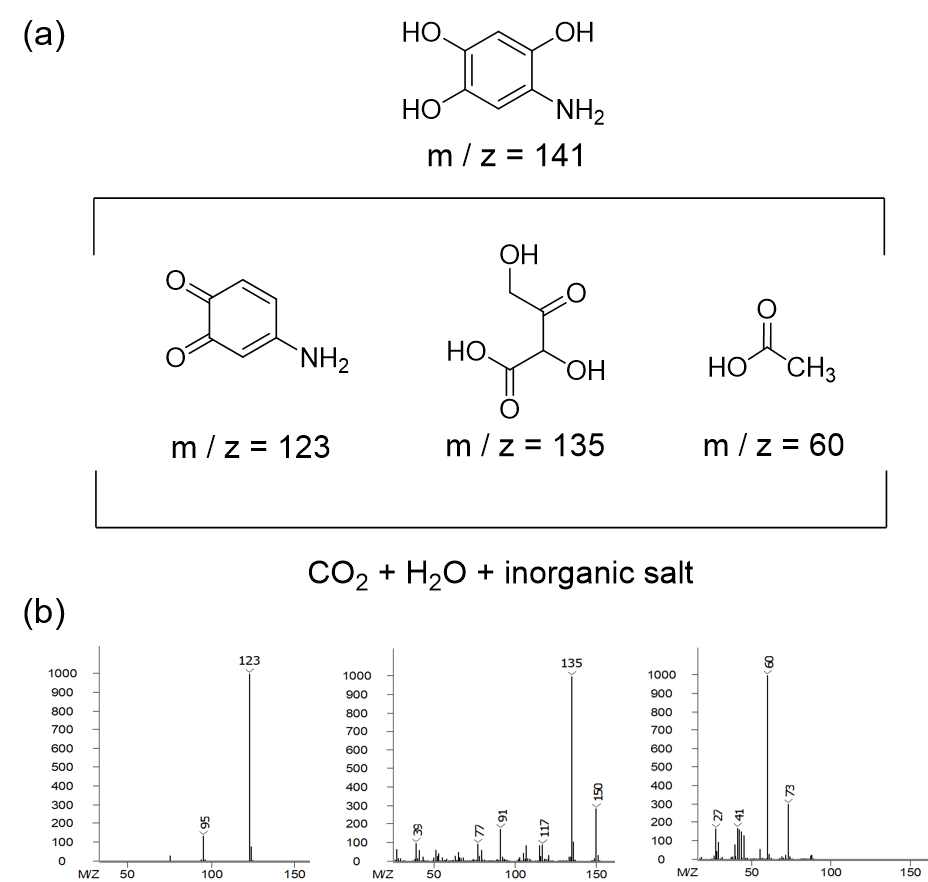


**Figure S31.** (a) Possible further intermediates derived from the polyhydroxy phenols intermediates obtained by P9 degradation in the Bi_12_FeO_20_-H_2_O_2_ system. (b) The corresponding GC-MS spectra of the m/z = 123, 135, and 60 intermediates.


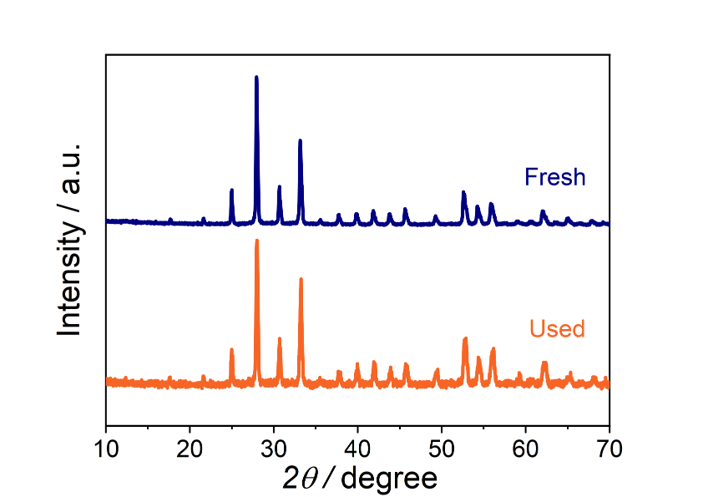


**Figure S32.** XRD patterns of fresh and used Bi_12_FeO_20_ nanosheets.


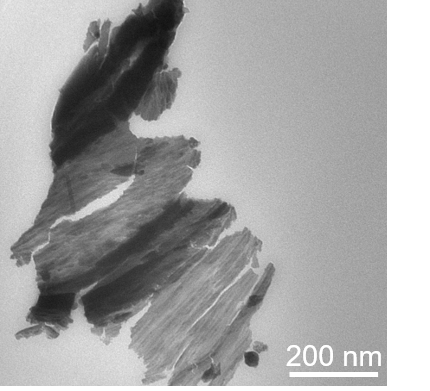


**Figure S33.** TEM images of Bi_12_FeO_20_ after repeated reaction cycles.

| Element | leakage/ppb | Proportion / % |
| --- | --- | --- |
| Bi | 61.177 | 0.0141 |
| Fe | 4.017 | 0.0410 |

**Table S6.** ICP-OES results of the Bi and Fe leakage of Bi_12_FeO_20_ into the solution after Fenton-like reaction for 100 min.


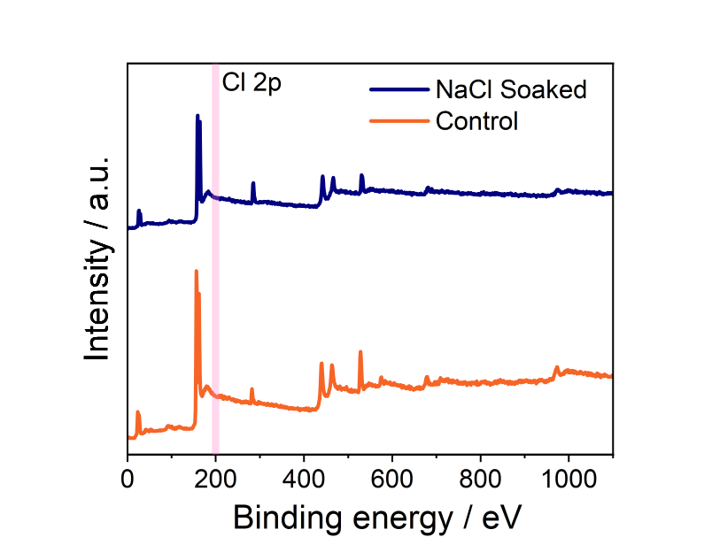


**Figure S34.** The XPS wide scan of Bi_12_FeO_20_ before and after soaking in NaCl aqueous solution for 48 h. [NaCl] = 1 M.


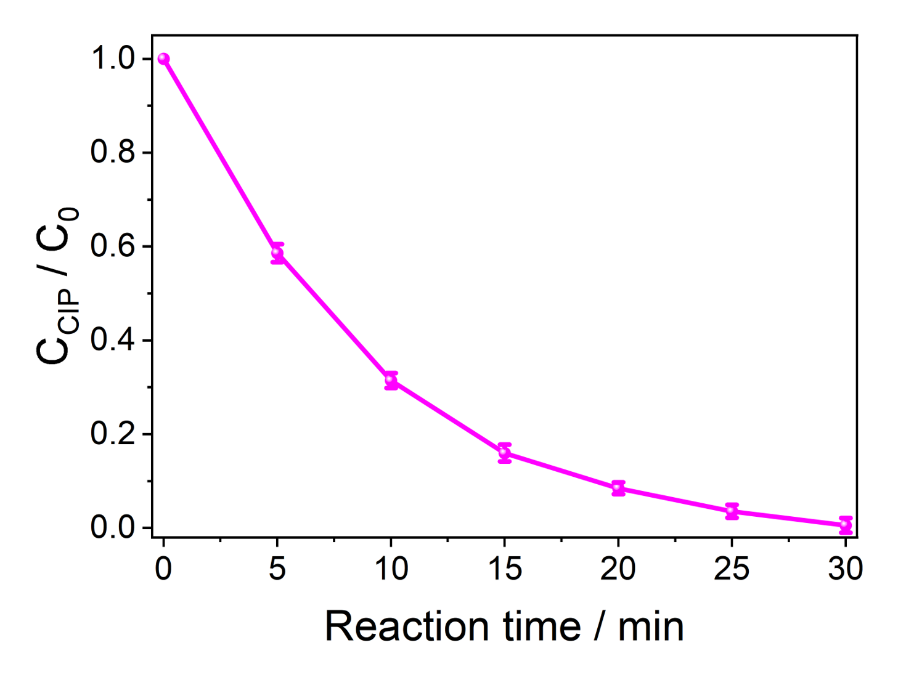


**Figure S35.** Degradation efficiency of CIP in pharmaceutical wastewater using the Bi_12_FeO_20_-H_2_O_2_ system. The pharmaceutical enterprise in Anhui Province, China, sampling date: December 12; CIP concentration: 3.7 mg L^-1^; pH: 8.38, underwent secondary wastewater treatment.

**References**

1 Z. Wang, Y. Wang, Y. Zhang, et al., "Efficient photothermal degradation on Bi12CoO20 sillenite with a strong internal electric field induced by the thermal effect," Applied Catalysis B: Environmental, 313, (2022): http://dx.doi.org/10.1016/j.apcatb.2022.121452

2 Y. Zhang, C. Pan, G. Bian, et al., "H2O2 generation from O2 and H2O on a near-infrared absorbing porphyrin supramolecular photocatalyst," Nature Energy, 8, (2023): 361-371. http://dx.doi.org/10.1038/s41560-023-01218-7

3 M. Huang, H. Z. Liu, Q. Q. Huang, et al., "Self‐Activated Heterogeneous Fenton Process for Accelerated Degradation of Aromatic Pollutants over Copper Oxide Catalysts," Angewandte Chemie International Edition, (2025): http://dx.doi.org/10.1002/anie.202508754

4 Z. Wei, S. Zhao, W. Li, et al., "Artificial Photosynthesis of H2O2 through Reversible Photoredox Transformation between Catechol and o-Benzoquinone on Polydopamine-Coated CdS," ACS Catalysis, 12, (2022): 11436-11443. http://dx.doi.org/10.1021/acscatal.2c03288

5 X. Wen, J. Miao, D. Mandler, et al., "Rotating ring-disk electrode method to evaluate performance of electrocatalysts in hydrogen peroxide activation via rapid detection of hydroxyl radicals," Chemical Engineering Journal, 454, (2023): http://dx.doi.org/10.1016/j.cej.2022.140312
